# Herbal products use during pregnancy and postpartum: study of consumption and user profile in Catalonia

**DOI:** 10.1186/s12906-025-05008-4

**Published:** 2025-08-08

**Authors:** Noelia G. Romero, Elisabet Teixido, Laia Guardia-Escote, Anna Tresserra, Salvador Cañigueral, Marta Barenys

**Affiliations:** 1 Unitat de Toxicologia-GRET, Departament de Farmacologia, Toxicologia i Química Terapèutica, Facultat de Farmàcia i Ciències de l’Alimentació, Universitat de Barcelona, Av. Joan XXIII 27-31, Barcelona, 08028 Spain; 2 Institute of Nutrition and Food Safety, University of Barcelona (INSA-UB), Barcelona, Spain; 3 Unitat de Farmacologia, Farmacognòsia i Terapèutica, Departament de Farmacologia, Toxicologia i Química Terapèutica, Facultat de Farmàcia i Ciències de l’Alimentació, Universitat de Barcelona, Av. Joan XXIII 27-31, Barcelona, 08028 Spain; 4 Polyphenol Research Group, Departament de Nutrició, Ciències de l’Alimentació i Gastronomia, Facultat de Farmàcia i Ciències de l’Alimentació, Avda. Joan XXIII, 27-31, Barcelona, 08028 Spain; 5 ZEBET, German Centre for the Protection of Laboratory Animals (Bf3R), German Federal Institute for Risk Assessment (BfR), Berlin, Germany

**Keywords:** Herbal medicine, Prenatal and postpartum health, Traditional use, Interviews, Consumption

## Abstract

**Background:**

The prevalence of herbal products (HPs) consumption among pregnant and postpartum women, the factors driving their use or the main sources of recommendation have never been studied in Spain or Catalonia. Investigating its prevalence of use during critical phases of development is crucial for providing guidance to health professionals.

**Methods:**

A validated questionnaire, containing general data on socio-demographic status, lifestyle, maternal health data and its association with HP consumption, was performed in online personal interviews among women living in Catalonia between pregnancy week 22 and postpartum month 9.

**Results:**

We identified a higher percentage of HPs consumption compared to other European countries, while the 5 most consumed products were similar to the products described to be consumed by pregnant women in other countries. The most frequently consumed HPs were ginger (28%), chamomile (9%), thyme (7%), rooibos (6%), cranberry (4%), and raspberry leaf (4%), and we identified specific temporal patterns of consumption for several of them, depending on the trimester of pregnancy. Furthermore, we found a significant relationship between women consuming oral HPs and the opinion that “pregnant women should preferably consume herbal remedies rather than conventional medicines”.

**Conclusions:**

We provide evidence that women consuming HPs during pregnancy are not defined by a specific profile and therefore, healthcare professionals should be aware that any woman could potentially consume HPs during this period.

**Supplementary Information:**

The online version contains supplementary material available at 10.1186/s12906-025-05008-4.

## Introduction

Nowadays, many people consume herbal products (HPs) for their health care in several regions of the world [1]. They do that for different purposes, including the prevention of various chronic diseases such as cancer, neurodegenerative and cardiovascular diseases or the treatment of symptoms and discomforts associated with respiratory, digestive or nervous disorders [2, 3]. In this study, HPs are defined as products containing herbal drugs, herbal drug preparations or their combinations as active ingredients, according to the definition given by Cañigueral et al., (2018) [4]. Briefly, on the one hand and based on the definition of the European Pharmacopoeia (Ph. Eur.), herbal drugs are mainly whole, fragmented or broken plants or parts of plants in an unprocessed state, usually in dried form but sometimes fresh. On the other hand, herbal drug preparations are homogeneous products obtained after subjecting herbal drugs to treatments such as extraction, distillation, expression, fractionation, purification, concentration or fermentation [5]. It is estimated that around 85% of the world’s population uses traditional medicines for their health needs [6]. The market for these products, which has expanded rapidly in recent years [7, 8], targets the general population, but the common designation of these products as “natural remedies” or “medicinal herbs” together with the belief that these products are “healthier” or “safer” than medicines makes some population subgroups with particular characteristics and a tendency to avoid the use of medicines, such as pregnant or lactating women, more prone to consume them. Several international studies reveal a high HPs consumption among pregnant population depending on the country, with 40% consumption rates in Switzerland, 44% in Australia and 69% in Russia [9].

In Catalonia, no study of these characteristics has ever been carried out, and therefore, the prevalence of consumption of HPs during pregnancy is not known, neither which are the most common consumption patterns during pregnancy or lactation (products, doses, time of consumption, or reasons). In fact, the only data available in Spain corresponds to the Telephone Information Service for Pregnant Women (SITE according to the Spanish acronym: Servicio de Información Telefónica para la Embarazada) which reported that among 156 pregnant women who inquired about consumption of infusions of different herbs during pregnancy, 42% had already ingested them before the query to SITE [10].

Many HPs are believed to have health-preventing or health-promoting effects and, therefore, are advertised as such [11]. However, there are cases of HPs that claim health benefits that are not scientifically proven [12]. During pregnancy, for example, supplements based on cranberry (*Vaccinium macrocarpon* Aiton) and rich in proanthocyanidins, are consumed with the aim of treating urinary tract infections, or supplements based on raspberry leaves (*Rubus idaeus* L.), rich in flavonols, anthocyanins and procyanidins are consumed with the belief that they can shorten and facilitate childbirth, but the effectiveness of these approaches is not clear [13, 14].

Despite being perceived as rather harmless and non-reactive products, HPs are composed of mixtures of compounds with a plethora of molecular targets such as protein-kinases, transcription factors sensitive to the reduction-oxidation status, cell cycle proteins and apoptotic and anti-apoptotic proteins. For example, some plant polyphenols, at high doses, have pro-oxidant activity [15], and in general, they are compounds capable of crossing the placental barrier [16, 17]. Also, some dietary polyphenols pass into human breast milk and therefore form part of the diet of newborns [18].

Because exposure during prenatal period and during the first months of life is of crucial importance for development, it is not only important to characterize the consumption of HPs during pregnancy, but also during lactation. This information will be useful for physicians and midwives, to understand which potential products their patients might use. This information will be valuable to identify which products require further research about their safety during pregnancy due to real exposure concerns.

By performing personal interviews to women between pregnancy week 22 and the 9th month postpartum, we aimed to determine the prevalence of HPs use among pregnant and breastfeeding women living in Catalonia. Our study focused on investigating the social characteristics of the participants, their reasons for using HPs, the specific products used and when they were used, who recommended their use, and the overall opinion of HPs consumption, particularly during pregnancy and breastfeeding.

## Materials and methods

The methodology used to achieve the aim of the study included the design of a questionnaire based on a previously published one, its validation and correction, the advertisement of the study to recruit volunteers, the performance of personal interviews online, the collection of the data and its statistical analysis.

### Questionnaire design

The questionnaire has been adapted from Kennedy et al. (2013), containing general data on socio-demographic status, lifestyle, maternal health data and its association with consumption of HPs during gestation and lactation [9].

### Questionnaire validation and Preparation of final questionnaire

The focused and guided interview model was selected, as the aim of the in-depth interviews is to obtain detailed information on the consumption of herbal products during pregnancy and lactation [19]. The Expert Judgement technique was used to validate the content of the questionnaire items [20]. This technique made it possible to determine whether the statements in the questionnaire are representative of the universe to be measured [21]. For each of the questionnaire items, the experts assessed three criteria: univocity, relevance and importance. Based on the validation of the questionnaire through the Expert Judgement technique, the statistical aspects of the 3 criteria evaluated: univocity, pertinence and importance (Figure S2-1), and the comments of the experts were considered to refine the final questionnaire, which can be found in Supplementary Material 1.

### Recruitment of volunteers

Information on participation in the study was disseminated through social media (Instagram, Facebook, Twitter), the GRET website, in medical care centers and various breastfeeding, parenting and childbirth preparation groups across the different health regions of Catalonia. These recruitment methods cannot avoid a potential selection bias towards women who are more familiar to use social networks, women who already have a high interest on caring about the health status during pregnancy or women who have a higher interest in the effects of HPs.

### Interview

The personal interviews were conducted in a virtual format using the Zoom (Version: 5.12.9) and Skype (Version 8.92.0.204) platforms due to the health emergency generated by SARS-CoV-2, thus respecting the recommendations of social distance and the different mobility restrictions applied during and after the state of alarm. The inclusion criteria were that the volunteers were resident in Catalonia (regardless of their country of birth) and were at least 22 weeks pregnant or had a child for a maximum of 9 months. Study participants were classified according to the health region to which they belonged within the Autonomous Community of Catalonia: Alt Pirineu and Aran, Lleida, Camp de Tarragona, Terres de l’Ebre, Catalunya Central, Girona and Barcelona [22]. Sample size was aimed at 100 interviews (number of respondents) taking into account that the number of pregnancies in Catalonia in 2019 was approximately 61,500 (https://www.idescat.cat/pub/?id=naix), considering a confidence level of 95% and aiming a margin error lower than 10%. With 102 valid respondents the final margin of error was 9.7%. As general descriptors of the study population in 2022 (or 2021 depending on latest data available), new mothers living in Catalonia had the following characteristics: mean age 32,6 years old; Spanish nationality 67%; maximum education level no education or primary school (14%), secondary school (14%), technical training (17%), high school (8%), university (42%), not known (12%), and they were distributed per province as follows: Barcelona 73%, Girona 10%, Lleida 6% and Tarragona 11%. The interview phase was between 30 July 2020 and 24 May 2023. Interviews were conducted in the same way with all participants, the same questions were asked and in the same order, while the time of the interviews was adapted to the needs of the women, including long breaks to attend the demands of the newborns, if needed.

### Data processing and statistical analysis

Data analysis was conducted using the Statistical Package for the Social Sciences (SPSS) version 27.0 software (IBM Corp, Chicago, USA). Descriptive statistics, including means and frequencies, were used to examine the different variables. Independent factors associated with the oral consumption of HPs during pregnancy and postpartum (with the dichotomous dependent variable: oral HPs consumption versus no oral HPs consumption) were assessed using a binary logistic regression. Subsequently, a Chi-square test was employed to further explore the significant factors, with the effect size (ES) evaluated using Cramer’s V coefficient. The graphs were generated using GraphPad Prism v.9. Results are presented as frequencies and percentages. Statistical significance was set at *p* ≤ 0.05.

## Results

### General characteristics of participants

**Fig. 1:**
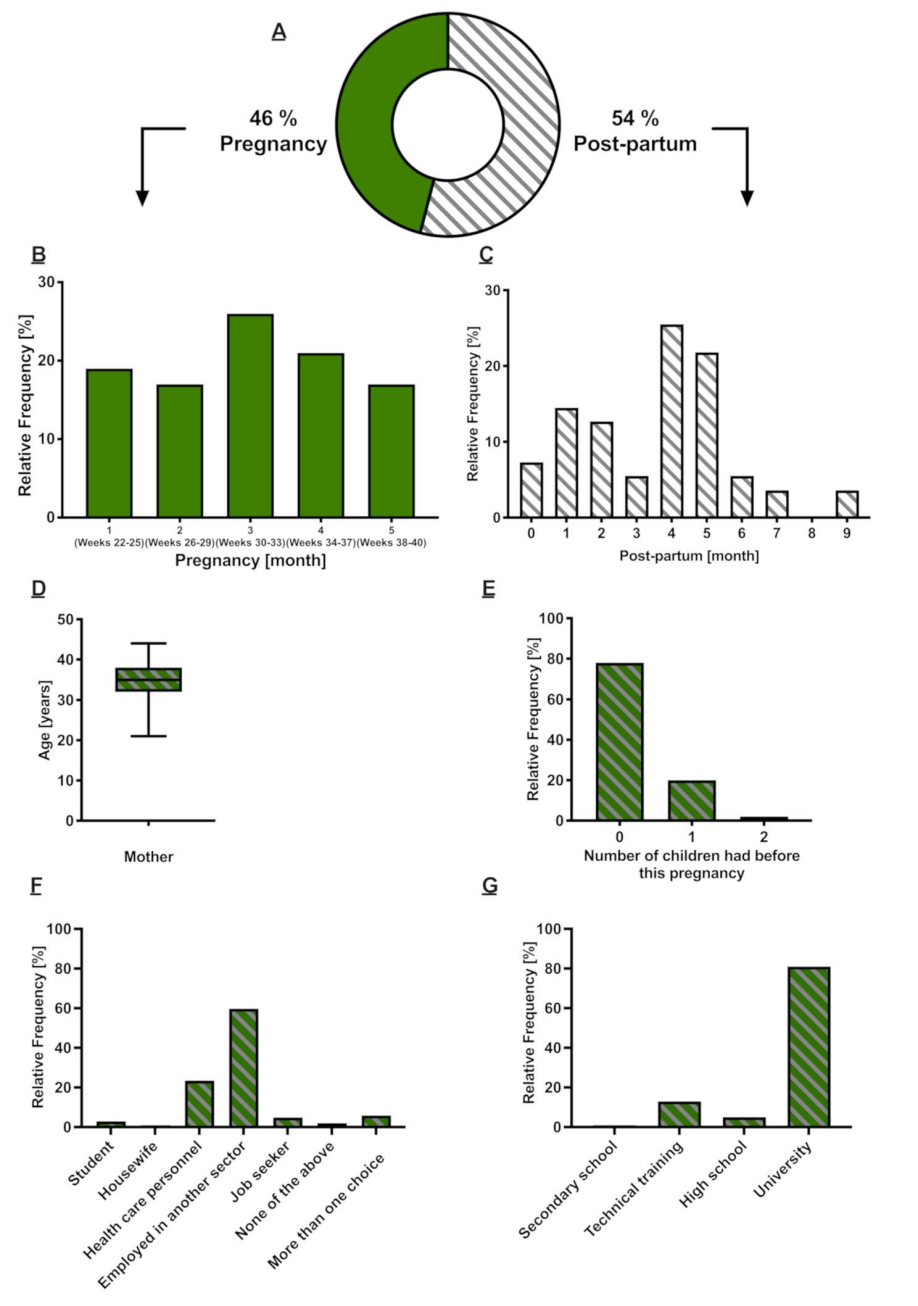
Profile of women interviewed (*n* = 102). (**A**) Women pregnant or postpartum at time of interview (%). (**B**) Months (and weeks) of gestation of pregnant women at the time of the interview (%). (**C**) Months postpartum of women at the time of the interview (%). (**D**) Average age of women who were pregnant or postpartum at the time of the interview (between 21 and 44 years). (**E**) Women respondents who reported the number of children they had before this pregnancy (%). (**F**) Employment status of interviewees at the moment of getting pregnant (%). (**G**) Highest level of education completed by the respondents (%)

A total of 106 interviews were conducted; however, four interviews were omitted due to lack of signature of informed consent, resulting in 102 valid interviews. Participants residing in Catalonia were recruited in 61 health centers with one or two participants from each center, except in six centers where three or more participants were recruited. Socio-demographic characteristics of women included in the study are presented in Table 1; Fig. 1.

**Table 1:** Socio-demographic characteristics of women living in Catalonia included in the study

		Frequency	Study sample [%] (*N* = 102)
Type of centre	Public	77	75
	Private	22	22
	Non-profit private	3	3
Healthcare region	Alt Pirineu i Aran	1	1
	Barcelona	67	66
	Camp Tarragona	10	10
	Catalunya Central	4	4
	Girona	9	8
	Lleida	7	7
	Terres de l´Ebre	4	4
Province	Barcelona	71	69
	Girona	9	9
	Lleida	8	8
	Tarragona	14	14
Country of birth	Spain	87	85
	Others	15	15
	Argentina	3	3
	Austria	1	1
	China	1	1
	Cuba	1	1
	Italy	2	2
	Paraguay	4	4
	Poland	1	1
	Dominican Republic	1	1
	South Africa	1	1

Among the women interviewed, 46% were interviewed during pregnancy and 54% during the postpartum (Fig. 1-A). The distribution of the frequency of interviews performed over-time can be observed in Fig. 1-B and C. The mean age of respondents was 34.6 years (range, 21– 44 years) (Fig. 1-D) and the median age was 35 years. The majority of the respondents were first-time mothers (78%), while 20% had already had 1 child and a smaller proportion had 2 children (2%) prior to this pregnancy (Fig. 1-E). At the time of becoming pregnant, most of the participants were working (24% in the healthcare sector and 60% in another sector; Fig. 1-F), while less than 10% were students, housewives or seeking a job. The surveyed women had mainly completed university studies (81%), and only a minority had technical training (13%), high school (5%), or secondary school (1%) as the highest achieved studies level (Fig. 1-G).

### Prevalence of HPs consumption

**Fig. 2:**
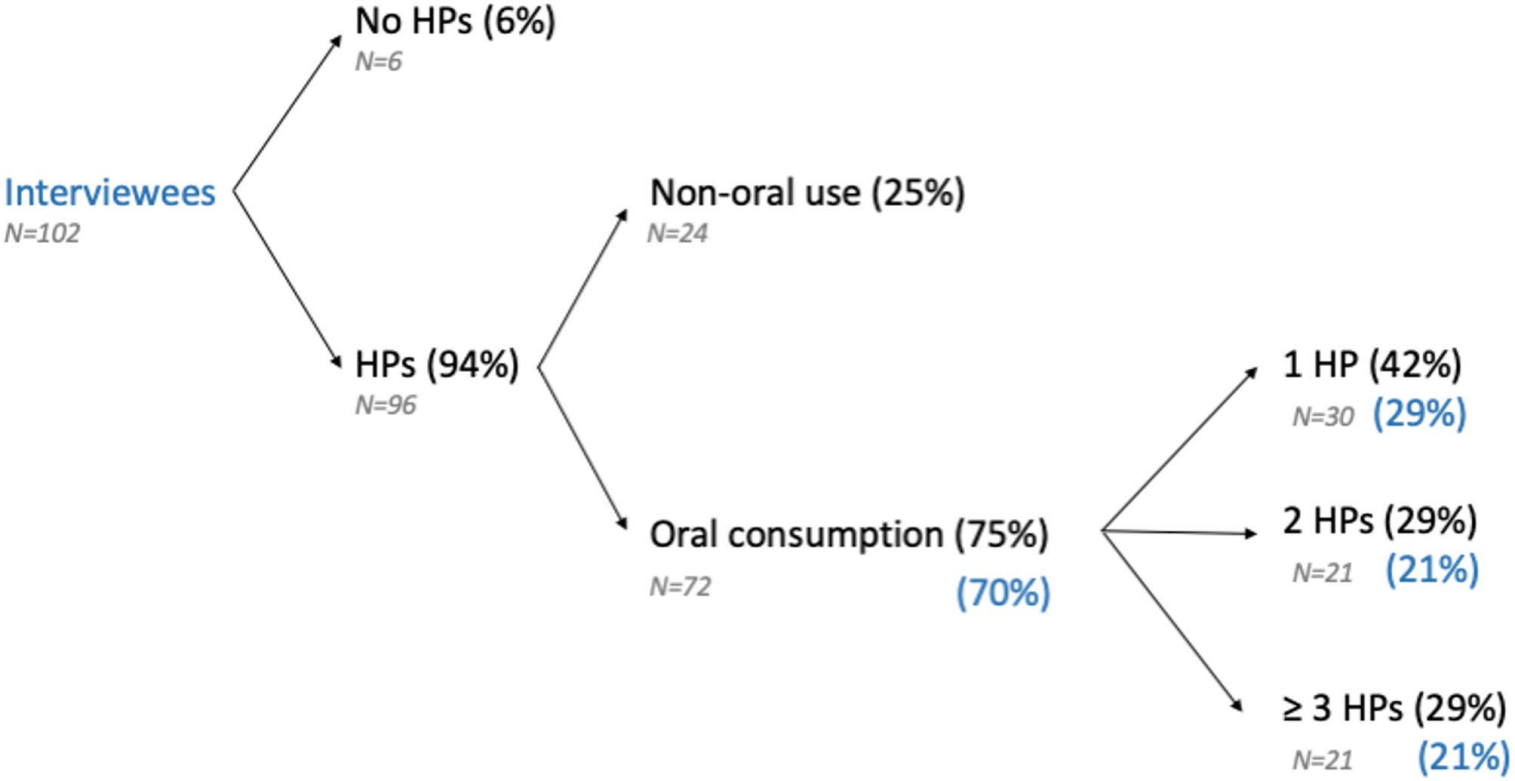
Results of consumption of HPs among the interviewees. Results of consumption during pregnancy and/or postpartum. Black percentages are calculated based on the number of interviewees included in the group at the arrow origin (specific N indicated in gray), blue percentages are calculated based on total respondents (*N* = 102). HP: herbal product

Among all study participants (*N* = 102), 94% (96/102) reported using HPs during their pregnancy and/or postpartum period, of which 75% (72/96) reported taking them orally, meaning that 70% of the participants consumed HPs orally (72/102). Within this group, 42% (30/72) reported using at least 1 HP, while 29% (21/72) reported using 2 HPs and another 29% (21/72) reported using more than 2 HPs at some point during pregnancy and the postpartum period (Fig. 2). In a more detailed evaluation, we distinguished between those reporting the use of HPs during pregnancy or not. The percentage did not vary greatly (Figure S2-2), meaning that 68% of interviewees consumed HPs orally during pregnancy, and 41% of interviewees consumed 2 or more HPs orally. Non-oral use included uses via dermal, nasal or rectal routes.

### Number, type and characteristics of HPs orally consumed

The total number of different HPs orally consumed at any moment (including pregnancy and/or postpartum), or consumed only during pregnancy was 165 and 155, respectively.

Independently of the time of consumption, the top ten orally consumed HPs were identified as follows (common name, scientific name most commonly associated to the common name; and percentage of consumption): ginger (*Zingiber officinale* Roscoe; 28%), chamomile (*Matricaria chamomilla* L.; 9%), thyme (*Thymus vulgaris* L. / *T. zygis* L.; 7%), rooibos (*Aspalathus linearis* (Burm. f.) R.Dahlgren.; 6%), cranberry/raspberry leaf/neoBianacid (4% each), floradix (3%), and milk thistle (*Silybum marianum* (L.) Gaertn.)/fennel (*Foeniculum vulgare* Mill.)/tea (*Camellia sinensis* (L.) Kuntze )/horsetail (*Equisetum arvense* L.) (2% each). Oral consumption of the remaining HPs represented less than 2% of the total for each product (Table S2-13).

The highest variability in types of HPs consumed was detected in the 1st trimester, the period considered as the most vulnerable to external exposures because it is when the main part of organogenesis occurs. Although there was this high heterogeneity in products consumed in the 1st trimester, a clear consumption pattern could be detected for certain HPs depending on the period of use (Table 2). Ginger stood out as the most consumed HP during the first and second trimester of pregnancy, with 61% and 21% consumption rates, respectively, and in some cases, it was consumed together with lemon juice as a flavoring masking agent. The third trimester was characterized instead by high consumption of raspberry leaves (30%). During the postpartum period, milk thistle was the most consumed HP (20%). For products reported to be consumed for longer periods than one trimester, once again, ginger stood out as the most consumed HP during 1st and 2nd trimester together; 2nd and 3rd trimester together; 1st, 2nd and 3rd trimester together and 1st, 2nd trimester and postpartum together. Rooibos on the other hand was the most consumed HP for the longest period possible to consider: the 1st, 2nd, 3rd trimester and postpartum together.

**Table 2:** HPs most consumed orally according to the period of consumption and ordered from shorter to longer consumption periods. The most orally consumed HPs are represented as a function of the total HP consumed in each period (frequency; %)

Consumption period^1^	Number of HPs	HPs most consumed
1st trimester	41	ginger (*n* = 25; 61%), chamomile (*n* = 3; 7%), cranberry (*n* = 2; 5%), thyme (*n* = 2; 5%).
2nd trimester	19	ginger (*n* = 4; 21%), thyme (*n* = 4; 21%), echinacea ((*n* = 2; 10%).
3rd trimester	23	raspberry leaves (*n* = 7; 30%), cranberry (*n* = 2; 8%), chamomile (*n* = 2; 8%).
Postpartum	10	milk thistle (*n* = 2; 20%).
1st and 2nd trimester	11	ginger (*n* = 2; 18%).
2nd and 3rd trimester	9	ginger (*n* = 3; 33%), chamomile (*n* = 3; 33%), neobianacid ^2^ (*n* = 2; 22%).
3rd trimester and postpartum	2	red fruits and ispaghula (*n* = 1; 50% each).
1st, 2nd and 3rd trimester	31	ginger (*n* = 7; 23%), chamomile (*n* = 5; 16%), rooibos (*n* = 4; 13%).
1st, 2nd trimester and postpartum	1	ginger (*n* = 1; 100%).
The participant cannot remember at which time-period she consumed the product	4	ginger, horsetail, pranarom calming^2^, tea (black, red or green) (*n* = 1; 25% each).

^1^HPs consumption frequencies in function of period are described in Tables S2-1, 11.^2^ Commercial herbal combinations (including ≥ 3 herbal drugs). Main herbal ingredients of commercial herbal combinations are listed in Table S2-12

**Fig. 3:**
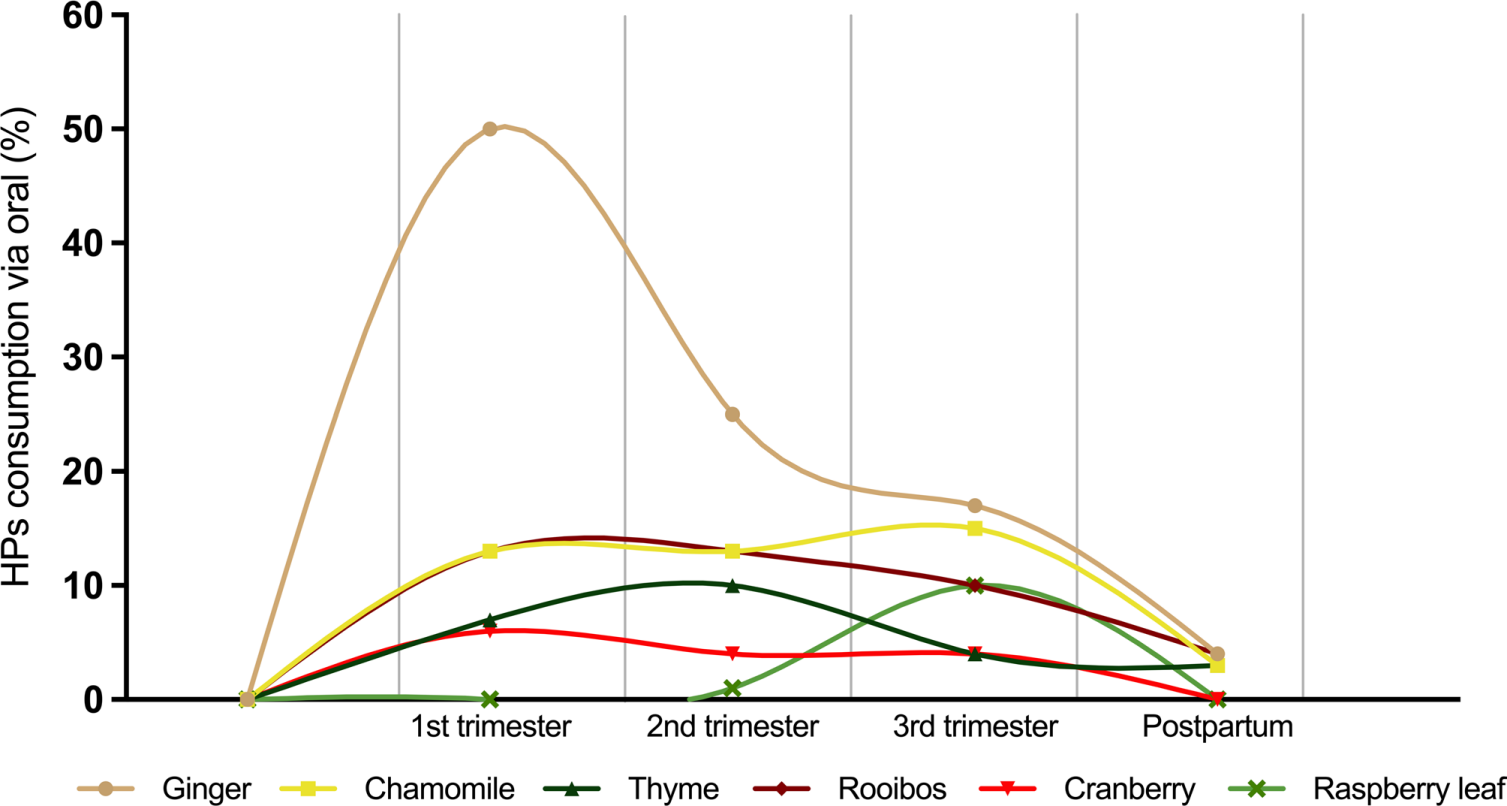
Prevalence of the six most consumed HPs by interviewed women who consumed oral HPs according to period of consumption: 1st, 2nd, 3rd trimester of pregnancy and postpartum (women who have had a child for at most 9 months). (Results presented in percentage of women consuming each of the six most consumed HPs for each trimester and postpartum out of the total number of women consuming HPs orally, independently on the duration of consumption). The lines connecting the data points are for visual representation purposes only and do not imply any predictions or trends in the data

Independently of wether the consumption was longer or shorter, the percentage of women consuming each of the six most consumed HPs in the study (ginger, chamomile, thyme, rooibos, cranberry and raspberry leaf) was calculated for each trimester and postpartum out of the total number of women consuming HPs orally (*N* = 72 from Fig. 2), to identify general trends of consumption over the whole period of study (Fig. 3). Ginger was standing out, with a notable peak of consumption in the first trimester (50% of oral HP consumers used it at least during the first trimester), maintaining relatively high levels of consumption even in the second (25%) and third trimesters (17%) of pregnancy; however, this use was decreasing clearly in the postpartum period (4%). Thyme was consumed more during the second trimester of pregnancy (10%), while rooibos was consumed more during the first and second trimesters (13%), raspberry leaf was only consumed during the third trimester (Fig. 3). The combinations of two HPs most commonly consumed together were the same ones in the first, second and third trimester: ginger-chamomile (with 9, 7 and 5% of the total number of combinations consumed in each trimester, respectively) and ginger-rooibos (with 7, 7 and 5%, respectively). During the postpartum there was no HP combination occurring more than two times.

To take into account that the moment during gestation or postpartum when the interview was performed could have an influence on the number of HPs orally consumed, simply because the later in gestation/postpartum the interview is conducted, the more time the person has had to consume HPs, we evaluated the mean number of products consumed depending on the moment of the interview and the number and proportion of women consuming HPs orally depending on the moment of the interview, but no significant effect was detected in any case (Figure S2-3).

The most common presentations chosen for oral consumption forms of the products reported in this study are detailed in Table 3. Infusion was the predominant form of consumption (62%), followed to a lesser extent by capsules (9%), with 8% consumption by tablets and syrups/fresh (6% each). Other presentations accounted for less than 5% of total consumption (such as dehydrated, powdered, drops, candies, oil, juice and cookies).

**Table 3:** Presentation of HPs most consumed orally

Presentation	Frequency	Percentage (%)
Infusion	103	62
Capsules	14	9
Tablets	13	8
Syrup	9	6
Fresh	9	6
Dehydrated	6	4
Powder	5	3
Droplets	2	2
Candy	1	1
Oil, juice, cookies	1	1

When considering all HPs in general, the main reason for their consumption was associated with nausea and gastric discomfort (21%), followed by incorporation into the diet (13%). Treatment and prevention of colds were also important reasons for consumption (11%), followed by use for stomach acidity (7%), along with relief of various types of pain and relaxing (4%), preparing for childbirth/stomach reflux/coffee substitute were less than 4% of the motives for consumption. Other reasons (less represented) for HPs consumption can be found in Table S2-14. The main reasons reported for consumption of the six most consumed oral HPs are detailed in Table 4.

**Table 4:** The six most orally consumed HPs and the main reasons reported by the participants for their consumption

HPs most consumed orally	Main alleged reasons for consumption	Frequency of women reporting each reason / total number of women who consumed the HPs.	Percentage of women reporting each reason (%)^1^
Ginger	Nausea, gastric discomfort	27/46	59
Chamomile	Stomach relaxant	4/15	27
	Pain (various)	3/15	20
	Nausea, upset stomach	3/15	20
Thyme	Cold (prevention and treatment)	7/11	64
Rooibos	Part of the diet	5/10	50
	Coffee substitute	3/10	30
Cranberry	Urinary infection (prevention and treatment)	5/7	71
Raspberry leaf	Preparing for childbirth	5/7	71

^1^Calculated in relation to the total number of women who consumed each of the HPs

In the next step we explored the origin of the recommendation/initiative for the consumption of HPs. Considering only women who consumed oral HPs (*n* = 72), more than half (63%) reported doing so on their own initiative, which might reflect common/popular knowledge about the HPs, otherwise, with such a high degree of true self-initiative, the patterns of consumption would be much more randomly distributed. We identified midwives as main external influencers of HP consumption during pregnancy, (47%), followed by recommendations by media (24%). Of note, in the Spanish system, midwives are normally responsible for conducting routine visits to women with low-risk pregnancies, and their recommendations for HPs, in most cases do not need approval by a physician. Friends also played a role in recommending HPs with 21% (Table 5), followed by pharmacists (19%). Regarding communication with their physician, half of the women (50%) informed their physician about the use of HPs, while 44% did not do so. Some participants (4%) indicated partial reporting (some products yes some not), while a minority could not recall whether they had informed their physicians or not (1%).

**Table 5:** Recommendation sources for the oral consumption of HPs during pregnancy and postpartum

Source	Frequency	Percentage (%) ^1^
Self-initiative	45	63
Midwife	34	47
Media	17	24
Others	16	22
Friends	15	21
Pharmacist	14	19
Family	12	17
Physician	9	13
Herbalist	4	6
Nutritionist	3	4
> 4 Options	3	4
Nurse	1	1

^1^Percentages add up to more than 100 as more than one source could be given

### Profile of HP oral consumers

Considering that a high percentage of women declare that they do not inform their physician about the HPs they consume orally, it would be desirable to obtain a profile of oral consumers of HPs for physicians to identify them and be able to ask and give advice in this regard in case it is considered necessary.

To obtain such a profile, the socio-demographic characteristics of the interviewed population were analyzed to find statistically significant characteristics associated with HPs oral consumption. Analyses of socio-demographic characteristics and oral consumption of HPs were conducted using a logistic regression, this initial analysis revealed a significant interaction (*p* = 0.004). The specific variables with significant association to HP oral use are health region (Exp(B) = 1.915, CI = 1.221–3.003, *p* = 0.005) and medicine consumption (Exp(B) = 5.009, CI = 1.328–18.894, *p* = 0.017). Although not statistically significant, a trend is observed in relation to employment status when the women became pregnant (Exp(B) = 0.634, CI = 0.380–1.058, *p* = 0.081) and presence of chronic pathology (Epx(B) = 3.010, CI = 0.910–9.961, *p* = 0.071), as presented in Table S3-1.

To measure the relevance of the only two significant parameters detected (health region and medicine consumption), chi-square analysis and Cramer’s V effect measure were performed. This analysis showed a weak association (ES = 0.195), between oral HPs consumption and health region, particularly between the Barcelona region and other health regions [χ² (1, *n* = 102) = 3.863, *p* = 0.049]. The chi-square analysis also identified a statistically significant association between oral HP consumption and medicine consumption [χ² (1, *n* = 101) = 5.549, *p* = 0.018; n is equal to 101 because one participant did not remember if she consumed medicines or not]. The impact of this relationship according to Cramer’s V test was considered moderate (ES = 0.234).

Folic acid consumption was also studied as a potential characteristic of such a profile, but separately from the socio-demographic characteristics. The consumption of folic acid was very high among the interviewees (98%), and no significant differences were observed in its consumption between the groups of HP users and non-users (98% vs. 100%), or between the groups of HP oral consumers and non-HP oral consumers (97% vs. 100%) by means of Fisher’s exact test.

### Consumers opinions on medicines and HPs in general and during pregnancy

We investigated women’s perceptions on the use of HPs in relation to the use of conventional medicines. This analysis aimed to determine whether the use of HPs was influenced by perceptions of their safety and efficacy, if they were considered safer and more natural than conventional medicines, or whether their choice was motivated by the ease of access compared to conventional medicines for the treatment of various ailments during pregnancy and postpartum. The questions asked (variables) were the same ones that were included in the questionnaire of Kennedy et al. (2013), and they are listed in Tables S3-2 and S3-3. Regarding the perception of medicine and HP use in general during life, to minimize the possibility of too many variables masking the results, the variables have been divided into more extreme, not extreme (Table S3-2) and positive, negative opinions (Table S3-3).

A binary logistic regression model revealed a significant association (*p* = 0.04) for responses considered as not extreme. Although a trend was observed between women taking oral HPs and the perception that health professionals could prescribe less medication if they had more time to interact with patients (Exp(B) = 1.563, CI = 0.942–2.593, *p* = 0.084), no significant relationship was found between oral HP use and any not-extreme response. On the other hand, the model showed no significance with respect to more extreme opinions (*p* = 0.078).

When classifying opinions into positive and negative, the model was found to be significant (*p* = 0.03) for positive opinions; however, no association was identified between HPs consumption and any individual opinion. In contrast, the model did not show significance for opinions categorised as negative (*p* = 0.089).

However, regarding the perception of medicine use specifically during pregnancy, women who consumed oral HPs clearly indicated that pregnant women should preferably consume herbal remedies rather than conventional medicines (Exp(B) = 1.862, CI = 1.133–3.060, *p* = 0.014) (Table 6).

**Table 6:** Women’s views on the use of medicines during pregnancy

Specific questions during pregnancy	B estimated coefficient	Standard Error	Wald statistic	Degrees of freedom	*p* value	Exp (B) Odds ratio	Confidence interval (95%)
I am more reluctant to take medicines when I am pregnant than when I am not pregnant.	0.429	0.362	1.407	1	0.236	0.651	0.320–1.323
Even if I am sick and can take medication, it is better for the foetus if I abstain from taking it.	0.198	0.278	0.505	1	0.477	0.821	0.476–1.416
Pregnant women should preferably use herbal remedies than conventional medicines.	0.622	0.254	6.008	1	**0.014**	1.862	1.133–3.060

Significance threshold established at *p* ≤ 0.05

We also explored women’s perceptions regarding the potential harm associated with consuming ginger and cranberry during pregnancy. Among all respondents (*N* = 102), the majority believed that ginger is not harmful (57%) or only not very harmful (31%). Similarly, 64% of respondents (*N* = 102) believe that cranberries are not harmful (Tables S3-4,5). This trend is consistent among respondents who have consumed these HPs. When women who have consumed ginger (*N* = 43) were asked about their perception of the safety of ginger, 70% of consumers believed that ginger was not harmful, followed by 23% who considered it to be not very harmful, and 7% who considered it to be partially harmful (Table 7).

**Table 7:** Opinions of the interviewed women who consumed ginger (for responses about ginger) or cranberry (for responses about cranberry) on their safety for the developing child

	Ginger		Cranberry	
Perception of safety for the foetus	Frequency	Percentage (%)	Frequency	Percentage (%)
Not harmful	30	70	5	83
Not very harmful	10	23	1	17
Partially harmful	3	7	0	0
Harmful	0	0	0	0
Very harmful	0	0	0	0
Unknown substance	0	0	0	0
Total	43^1^	100	6^2^	100

^1^Based on the total number of interviewees who consumed ginger. ^2^Based on the total number of interviewees who consumed cranberry

In the case of cranberry consumers (*N* = 6), 83% share the opinion that the consumption of cranberries is safe, while the remaining 17% believe it is not very harmful (Table 7).

## Discussion

This study represents the first analysis carried out in Catalonia that addresses the prevalence of consumption of HPs by women during pregnancy and postpartum. Previous research on the use of medicinal plants in Spain has been mainly based on their traditional application or the prevalence of consumption in the general population. However, there is a need to acquire more knowledge regarding the real consumption of HPs during these specific stages in life.

Our research shows that a very high percentage of Catalan pregnant and postpartum women consume HPs orally (70%). This prevalence of use exceeds the one documented for pregnant women in other European countries such as France, the Netherlands, Austria, and Switzerland, where use has been reported at 16, 16, 38 and 41% respectively [9], but is similar to prevalences reported in a small study for general population in Barcelona (60%) [23] or in Madrid (70% occasional users with 1–4 times a month, 10% habitual users with > 4 times a month) [24], or higher than in a partial study on the general population in Catalonia (50%) [9, 25]. This result could represent a similar very high prevalence of HP consumption in pregnant women than in the general population, but could also be related to the fact that several studies have already pointed out a higher HP consumption among women than among men [24, 26]. Also, most of the participants in our study had a high level of education (81%), and most of them had a job at the time of getting pregnant (84%). These percentages are clearly above the percentage of Catalan population between 25 and 34 years old having a high level of education in Catalonia (57%, Data from year 2022, source IdesCat: https://www.idescat.cat/indicadors/?id=ue%26n=10100), and above the employment rate among women in Catalonia (50%, Data from 2022, idescat: https://www.idescat.cat/indicadors/?id=basics%26n=10218&t=202204) [27, 28]. With this context we can see that, women with a high level of education and with a job were overrepresented in our study, and this overrepresentation could be responsible for the high percentages of HP consumption we have detected. The differences in prevalence between studies may also be attributed to variations in the definitions of HP, local practices, and specific population characteristics such as education levels and target groups (e.g., pregnant women) and forms of use/consumption (oral or topical). We cannot exclude the possibility that women who were consuming HPs regularly may have been more inclined to participate in our interviews due to their higher interest in the effects of HPs. We also have to take into account that HPs consumption has been associated with high educational achievement and with employment status (being employed) [29, 30], characteristics that are present in high proportion in our sample.

Regarding the number of HPs consumed per woman, our results were also higher than in the specific literature for pregnant women in other countries: other authors have reported a mean consumption of 1.2 and 1.7 HP/woman during pregnancy [31, 32], while we report that the mean consumption was 2.3 HP/woman during pregnancy and postpartum. If the reasons behind this higher consumption are also related to the higher consumption patterns in the general population needs further investigation, since the local studies found do not report the number of HPs consumed per person.

The first trimester of pregnancy was the period with the highest variety of HPs consumed (*N* = 41) with a big difference compared to the next two time periods with highest variety: combination of the three trimesters (*N* = 31), and the third trimester (*N* = 23). This trend could be attributed to several factors: it is possible that in the early stages of pregnancy women are not aware of their condition, and this leads to unintentional exposures during pregnancy, that are stopped as soon as they get aware of their pregnancy. Another potential explanation is that there is a higher incidence of pregnancy-related health problems/discomforts during the first trimester, which could lead to an increase in the consumption of these products [33]. However, it is also important to mention, that data consumption could be skewed to the earlier pregnancy months in our study because women were interviewed once from gestational week 22 until postpartum, and therefore, all women can report about consumption during the early pregnancy months, but not all can report about very advanced pregnancy stages or postpartum.

For each of the six most consumed HPs in general, we discuss their consumption following the structure: HP consumption in our study; literature about consumption of this HP among pregnant women; information about its efficacy for pregnancy related discomforts; information about its safety during pregnancy-lactation; recommendations of the European Medicines Agency (EMA) or European Scientific Cooperative on Phytotherapy (ESCOP), if available.

The most consumed product to relieve these discomforts was ginger. Nausea affects approximately 70–80% of pregnant women, with around 50% experiencing additional vomiting [34–36]. Ginger consumption has been documented to be associated with relief of nausea and vomiting during pregnancy, therefore it is not surprising that in our study 59% of women who consumed ginger orally indicated that they did so primarily to treat vomiting and gastric discomfort. Kennedy et al., (2013) conducted a multinational study and showed that ginger was one of the commonly consumed HPs with nausea and gastrointestinal disorders being the most common reason for consumption [9]. In addition, episodes of nausea and vomiting during pregnancy have a significant negative impact on women’s lives [37]; in fact, Heitmann et al. (2017) reported that 76% of women with severe symptoms of nausea and vomiting considered not becoming pregnant again [38]. Clinical studies using ginger to treat nausea and vomiting during pregnancy have reported a significant improvement in nausea symptoms [39, 40] or also an additional significant decrease in vomiting [39]. Scientific studies have found ginger to be effective in treating nausea and vomiting in pregnancy, showing comparable and even superior results to vitamin B6 and dimenhydrinate [41, 42]. The EMA advisors, based on these and related studies comment that overall, there is sufficient scientific documentation to support the efficacy of ginger root for pregnancy-induced nausea and vomiting [43]. However, interactions of ginger with drugs commonly used during pregnancy, such as insulin, metformin and nifedipine, have been identified [44], and also some studies have also linked ginger consumption during pregnancy with a significant decrease in gestational age at delivery and neonatal head circumference [45]. One of the main critical control points to ensure safety and quality of ginger products is its chemical composition (active components like gingerols, or potentially toxic ones like methyleugenol), therefore it would be relevant to monitor the concentration of constituents like methyleugenol [46]. In view of all these aspects, and following the pattern of traditional use, the EMA advises caution in the use of ginger during pregnancy, suggesting to avoid its consumption as a preventive measure. Also, due to insufficient data, its use is not recommended during lactation [47]. ESCOP states that during pregnancy and lactation no data is available and according to general medical practice, the product should not be used during pregnancy and lactation without medical advice [48].

The second most consumed product was chamomile. This result agrees with a previous study conducted in Italy with 630 women who had given birth revealing that chamomile was the most commonly used HP (37.5%) [45]. The benefits of using chamomile are due to the fact that the essential oils and flower extracts derived from chamomile contain more than 120 chemical components, many of which are pharmacologically active [49]. Modares et al., (2012) conducted a study involving 105 pregnant women with mild to moderate symptoms of nausea and vomiting. The results showed that the Rhodes index score, which assesses symptoms of vomiting and nausea, was significantly lower in the group receiving chamomile compared to the placebo group (*p* < 0.05) [50]. However, chamomile contains coumarin derivatives, which could pose a potential risk for women with coagulation disorders during pregnancy [51]. Cuzzolin et al., (2010) found that 21.6% of women who used chamomile regularly during pregnancy experienced a higher incidence of threatened miscarriage and preterm birth compared to those who did not [52]. Due to a lack of evidence for safety and efficacy and to the risk of allergic reactions, chamomile is generally not recommended during pregnancy [53]. Therefore, its traditional use during pregnancy and lactation has not yet been recommended by the EMA [54], while ESCOP states that no harmful effects associated with chamomile consumption during pregnancy and lactation have been reported [55].

Thyme has traditionally been used to treat coughs associated with colds. We observed that 70% of users who consumed thyme consumed it primarily to treat or relieve cold symptoms. Raoufinejad et al., (2020) found in a hospital survey of 325 women in Iran that thyme was one of the most widely consumed herbal products (17%), the main reason for oral and inhalation use was for the treatment of infections [56]. Research into the effects of thyme consumption during pregnancy on prenatal exposure endpoints in pregnant test animals and developing organisms is crucial, as recommended by the Organization for Economic Co-operation and Development guidelines on prenatal developmental toxicity study. This includes assessing maternal effects, as well as fetal outcomes such as death, structural abnormalities, or altered growth [57]. Moreover, it is essential to determine the maternal/fetal effluent, indicating the placental transfer of suspected chemicals or metabolites. In a study with pregnant rats, postmortem examinations were carried out to measure the concentration of thymol sulphate (TS) in maternal and fetal effluent extracts. The study found that TS levels increased in both mothers and fetal tissue depending on the dose administered. Additionally, TS was found to cross the placental barrier in rats. Notably, administering high and repeated doses of thyme extract resulted in an increased resorption index. This study highlights the potential risk of exceeding certain doses when using thyme remedies during pregnancy [58]. A study by Zaineh et al., (2020) revealed that high doses of thyme extract and its chronic use in pregnant rats can alter placental functions, affecting foetal growth [59]. However, due to insufficient data, the EMA has not yet established the safety of thyme consumption during pregnancy and lactation [60]. Similar to the EMA recommendation, ESCOP recommends that as no data on safety during pregnancy and lactation are available and in accordance with general medical practice, the product should not be used during pregnancy and lactation without medical advice [48].

Research has shown that cranberry is one of the commonly used plants during pregnancy. During pregnancy, its use focuses on the prevention or treatment of urinary tract infections and vaginal candidiasis [9]. In a Norwegian cohort study, which included data from more than 100,000 pregnant women in 1999–2008, the use of cranberry was reported, and this study revealed that 919 (1.3%) had used cranberry during pregnancy. Of these women, 61.6% had used cranberry in early pregnancy, and 60.3% had experienced urinary tract infections [61]. These findings are comparable with the results obtained in our study, where 71% of women who used cranberry did so to prevent and/or treat urinary tract infections. Urinary tract infections (UTIs) are the most common bacterial infection during pregnancy and they increase the risk of maternal and neonatal morbidity and mortality [62]. In addition, UTI during pregnancy has been associated with an increased risk of pre-eclampsia (PE) and preterm birth. On the other hand, maternal UTI has also been associated with an increased risk of epilepsy in the children of women without epilepsy, although the association is weak. Maternal UTI has also been linked to an increased risk of attention deficit hyperactivity disorder (ADHD) [63–66]. Some studies have suggested that cranberry juice or cranberry supplements may reduce the incidence of UTIs in healthy women [67]. In vitro research results show that cranberry-derived compounds, such as polyphenols and type A proanthocyanidins, may interfere with bacterial adhesion to urinary tract epithelial cells, reduce the reservoir of uropathogens in the gastrointestinal tract and suppress the inflammatory cascade [68]. A meta-analysis by Fu et al., (2017) suggests that cranberry may represent a non-pharmacological approach to prevent the recurrence of uncomplicated urinary tract infections in generally healthy women. It has been demonstrated that cranberry concentrate contains a high level of oxalate, which could increase the risk of urinary tract stone formation in patients with a history of lithiasis [69]. Therefore, its use is not recommended in pregnant women, as urinary tract symptoms in this population group require medical supervision, and due to insufficient data, the use during pregnancy and lactation is not recommended [70, 71].

Raspberry leaves are believed to have uterotonic effects. Recently, it was shown that up to 25% of 578 pregnant women in a British hospital antenatal clinic had used raspberry leaves [31]. In addition, a national survey of 500 American College of Nurse-Midwives members revealed that 63% of certified nurse-midwives used raspberry leaf to stimulate labour. The commonly cited reason for using herbal preparations for this purpose was their “natural” nature, while those who did not use them were due to a lack of research or experience on the safety of these substances [72]. Several sources on internet and other media often recommend the consumption of raspberry leaves during pregnancy to stimulate and facilitate labour and shorten its duration. However, according to Zheng et al., (2010) the assessment of the direct effects of various commercial red raspberry leaf preparations in vitro on the contractility of uteri collected from non-pregnant and late pregnant rats treated with diethylstilbestrol could not confirm these statements. These products sometimes increased the effect of oxytocin and sometimes caused an increase in contraction followed by inhibition and he concluded that the biological activity of red raspberry leaf preparations varies depending on the herbal preparation used and the stage of pregnancy. These results do not support the hypothesis that red raspberry leaf preparations facilitate labour by a direct effect on uterine contractility [73]. According to recent studies, the consumption of raspberry extracts may be associated with the occurrence of hypoglycemic episodes, which is a dangerous complication, especially in women with gestational diabetes [74]. In addition, the substances contained in raspberries can have an impact on coagulation. Studies have shown that ellagic acid (which is present in a high proportion in raspberries) has a significant hyper-coagulant effect; the use of ellagic acid has been associated with shortening clotting times and increasing thrombin activity [75]. Therefore, the EMA has not yet established the safety of raspberry leaves during pregnancy and lactation, although they are still widely consumed [76].

Rooibos was also classified as one of the most widely consumed HPs in our study. The main reasons reported by consumers of this herb are that they consume it as part of their diet (50%) and as a coffee substitute (30%). The latter reason is attributed to its popularity as an herbal tea, mainly due to the absence of alkaloids such as caffeine, making it appear as a healthy beverage option [77]. Since the consumption of this HP responds more to recreational reasons than to health reasons, there is less data regarding the potential beneficial or adverse effects during pregnancy, and there are no comments from the EMA about its use during pregnancy. In case it is of interest to readers focusing on HP consumption only due to health reasons, we have calculated if the percentage of population consuming HPs orally would have changed in case rooibos would have been excluded from the analysis. In this case, there is no variation in the total percentage of oral HP consuming women because all women who consumed rooibos also consumed other oral HPs (Fig. 2).

Observing the opinions on the safety of HPs among our participants and comparing them to the statements and recommendations of EMA and ESCOP for the different HPs we have researched, we can conclude that they are not aligned, and that the high perception of safety identified for some products is not based on statements of official organizations.

In general, in our study, the main route of administration was by oral consumption, with infusions being the most common form used by women, prepared from dried or fresh plants. It is crucial to recognise that the form of administration of HPs can influence the bioavailability and therefore the therapeutic efficacy of the active compounds present in the infusions or the appearance of adverse effects. For example, research such as that conducted by Kubra et al., (2012), has shown that the chemical content of ginger can vary significantly depending on factors such as place of origin and whether the rhizomes are fresh or dried. In addition to infusions, there are other forms of HPs consumption, such as tablets, pills and syrups, which are often available over the counter [78]. According to the literature, in Spain HPs are acquired in pharmacies, but also in supermarkets or herbal shops [23, 24], however, by legal regulation, those HPs classified as herbal medicines can only be acquired in pharmacies. Of concern is that these products are marketed as dietary supplements without clear specification of dosage, active constituents and/or declaration of warnings for consumption during pregnancy and lactation, which poses significant challenges in terms of the safety and efficacy of these products [79]. Moreover, a significant proportion of women consuming them do not report that consumption to their medical doctors, and then no specific additional information/recommendations can be given to them.

The primary source of recommendation to orally consume HPs was self-initiative, which is consistent with Cuzzolin et al., (2010) where self-initiative was also the main source (34.6%), followed by parents/friends/media, which together accounted for 33.3% [52]. However, these recommendation sources in our study accounted for 17%, 21%, and 24%, respectively, which is higher and more comparable to a small study in Barcelona indicating familiar tradition and friends as recommending sources in 43% and 22% of cases (familiar tradition in this case includes self-initiative and parents/other relatives’ recommendation). A 13% share of doctors as a source of recommendation was observed in our study; which is consistent with lower percentages (4%) reported by general population in Barcelona [23], however, women reported that the main recommendation, after self-initiative, came from midwives (47%), which is evidence of a substantial influence on women’s behaviour regarding the use of HPs [80]. A survey of 309 midwives and midwifery students from 7 midwifery schools across Germany reported that 30% of the modality they recommended was towards herbal medicine. Similarly, in another survey of certified midwives in North Carolina, USA, out of 82 participants, 74% recommended that women use herbal medicine treatments [80]. The fact that midwives recommend HPs during pregnancy and lactation may be mainly influenced by their personal use of HPs, which leads them to recommend them to women [81].

To offer a better context to the results of our study, it would be desirable to have information on the percentage of oral consumption of HPs in the population of non-pregnant/non-postpartum Catalan women, but to the best of our knowledge this data separating men and women, is not available. To have an approximation to this information, we have obtained the raw data from an official survey of the Catalan Agency of Food Safety (ACSA) regarding the consumption of food supplements in the Catalan population. According to this data [82], there is a significant association between the consumption of food supplements and sex in the general Catalan population: 28.3% of men consume these supplements, compared to 35% of women. Analysis of the frequency of supplement consumption by age range reveals a higher consumption pattern in women than in men in the 25–34 age range (Figure S3-1). Searching for information at a Spanish level for comparison, in a study by Sánchez et al., (2020), which assessed 534 individuals in the Autonomous Community of Madrid, the most common pattern of HPs use was among young women aged 18–44 with higher education [24]. Both results would be consistent with our higher percentage of HPs consumption among women than the reported ones for partial studies in general population in Barcelona, Madrid or Catalonia.

A limitation of our study is the relatively low number of participants, however other studies with similar or lower numbers of participants in populations of similar number of inhabitants have been performed [9]. Related to this low number of participants, our study population is representative of women with a higher educational level than the general population. To improve generalizability, future research should aim to include more women with lower educational levels, ensuring a more balanced representation. Nevertheless, the main strength of our work is the use of face-to-face interviews focused on detecting the use of HPs and understanding the motivations behind women’s use of HPs. The main advantage of this approach lies in obtaining a wide range of information without leaving room for possible confounding. The quality of the data obtained with this approach is considered to be much higher than the one obtained with written form questionnaires online, but it consumes a much higher amount of time, and therefore, a lower number of participants can be obtained.

Despite this limitation, this study provides for the first time a detailed overview of the consumption of HPs during pregnancy and postpartum in Catalonia. It has the advantage that it includes participants with a balanced representation among the whole territory, with women from 61 different health centres in Catalonia. Asking not only about the consumption facts, but also about the perceptions and opinions behind the consumption of HPs is also a strength of this approach that helps to obtain a better context and to understand better the situation. It also provides detailed information on the main routes of consumption, the most consumed HPs, the reasons behind their consumption and the main sources of recommendations. Our results highlight the need for further research on the safety of these products during these critical stages of human development.

## Conclusions

Our study provides a comprehensive analysis of herbal product (HP) consumption among pregnant and postpartum women in Catalonia, revealing a high prevalence of use. The findings highlight that HP use is not limited to a specific demographic profile, emphasizing the need for healthcare professionals to proactively inquire about HP use among all patients during pregnancy and lactation. The most commonly consumed HPs-ginger, chamomile, thyme, rooibos, cranberry, and raspberry leaf-were often used for managing common pregnancy-related symptoms such as nausea, gastrointestinal discomfort, and urinary tract infections.

While the frequent use of HPs reflects a strong cultural acceptance of natural remedies, it also raises concerns related to the lack of scientific evidence supporting the safety and efficacy of certain products during pregnancy and postpartum. The study stresses the importance of educating both healthcare professionals and women about the potential risks and benefits of HP consumption, particularly in the absence of clear regulatory guidelines or safety data for use during critical developmental stages. Furthermore, it calls for integrating information about HP use into routine prenatal and postnatal care, fostering open communication between patients and healthcare providers to ensure informed decision-making.

## Electronic supplementary material

Below is the link to the electronic supplementary material.

Supplementary Material 1

Supplementary Material 2

Supplementary Material 3

## Data Availability

Data described in the manuscript, code book, and analytic code will be made available upon reasonable request to the corresponding author.

## Abbreviations

ACSA: Catalan Agency of Food Safety
ADHD: Attention deficit hyperactivity disorder
EMA: European Medicines Agency
ES: Effect size
ESCOP: European Scientific Cooperative on Phytotherapy
GRET: Grup de Recerca en Toxicologia
HP: Herbal product
PE: Pre-eclampsia
SE: Standard error
SITE: Spanish Telephone Information Service for Pregnant Women
SPSS: Statistical Package for the Social Sciences
TS: Thymol sulfate
UTI: Urinary tract infection
WHO: World Health Organization
