# Supplementary material for: Herbal products use during pregnancy and postpartum: study of consumption and user profile in Catalonia
Source: BMC Complement Med Ther. 2025 Aug 8;25:301. doi: 10.1186/s12906-025-05008-4 (PMC12335089; doi:10.1186/s12906-025-05008-4)
Supplement: Supplementary file 1 — Supplementary Material 1 [file 12906_2025_5008_MOESM1_ESM.docx]

***RESEARCH QUESTIONNAIRE***

Consumption of herbal products (HPs) during pregnancy and breastfeeding

- Toxicology Unit -

- Faculty of Pharmacy and Food Sciences

- University of Barcelona -

**Code: ____________**

**Recruitment Centre: ________________**

**PERSONAL INFORMATION**

| **1. In which Autonomous Community do you live? 2. In which Province? 3. What is your country of birth?** | |
| --- | --- |
| **2. Are you pregnant right now?**  □ Yes □ No | |
| **If answer 2 is yes:**  **What week of pregnancy are you in?**  □ 0 to 19  □ 22 to 44 | **If answer 2 is negative:**  **How old is your last child (in weeks/months)?**   - 0-4 weeks /0 month - 4-8 weeks / 1 month - 9-12 weeks / 2 months   □ 13-16 weeks / 3 months  □ 17-20 weeks / 4 months  □ 21-24 weeks / 5 months   - More than 6 months |
| 1. **How many children did you have prior to this pregnancy?**    - None    - 1 (one)    - 2 (two)    - More than two | |
| 1. **What is the highest level of education you have completed?**  \| □ Primary education (PE) \| □ High school (HS) \| \| --- \| --- \| \| □ Compulsory Secondary Education (ESO) \| □ University \| \| □ Vocational training (VT) \|  \| \| □ Other education________________________ \|  \| | |
| 1. **What was your employment status when you became pregnant? You can check more than one option**    - Student    - Housewife    - Healthcare worker    - Employed in another sector    - Job seeker    - None of the above   If your answer is the option ¨Worker in the health sector¨ or ¨Employed in another sector¨  Specify profession: ___________________ | |
| **6. Age:** | |
| **PREGNANCY INFORMATION**   1. **Have you taken any folic acid supplements (alone or as part of multivitamins)?**    - Yes, before pregnancy    - Yes, before and during pregnancy    - Yes, only during pregnancy    - No    - I do not remember   **Which one(s)? ______________________________________________ (show pictures of packaging)** | |

| **HEALTH DISORDERS AND PRODUCTS/MEDICATIONS CONSUMED DURING PREGNANCY**   1. **Use the Table to answer the following questions:**    1. **During this pregnancy have you experienced any of the disorders listed below?**    2. **Do you use or have you used any medications in connection with [each health condition listed]? Which one?**    3. **In what weeks of pregnancy have you used it/them?** | | | |
| --- | --- | --- | --- |
| **Disorder** | | **Medication** | **Period of use (week/month/trimester of pregnancy)** |
| Nausea | - Yes - No |  | - Week 0-15 / 1-3 months / 1st Trimester - Week 16-28 / 4-6 months / 2nd Trimester - Week 29-delivery / 7-9 months / 3rd Trimester - Postpartum - I do not remember |
| Heartburn or reflux problems | - Yes - No |  | - Week 0-15 / 1-3 months / 1st Trimester - Week 16-28 / 4-6 months / 2nd Trimester - Week 29-delivery / 7-9 months / 3rd Trimester - Postpartum - I do not remember |
| Constipation | - Yes - No |  | - Week 0-15 / 1-3 months / 1st Trimester - Week 16-28 / 4-6 months / 2nd Trimester - Week 29-delivery / 7-9 months / 3rd Trimester - Postpartum - I do not remember |
| Common cold | - Yes - No |  | - Week 0-15 / 1-3 months / 1st Trimester - Week 16-28 / 4-6 months / 2nd Trimester - Week 29-delivery / 7-9 months / 3rd Trimester - Postpartum - I do not remember |
| Urinary tract infections | - Yes - No |  | - Week 0-15 / 1-3 months / 1st Trimester - Week 16-28 / 4-6 months / 2nd Trimester - Week 29-delivery / 7-9 months / 3rd Trimester - Postpartum - I do not remember |
| Other infections | - Yes - No |  | - Week 0-15 / 1-3 months / 1st Trimester - Week 16-28 / 4-6 months / 2nd Trimester - Week 29-delivery / 7-9 months / 3rd Trimester - Postpartum - I do not remember |
| Muscle pain | - Yes - No |  | - Week 0-15 / 1-3 months / 1st Trimester - Week 16-28 / 4-6 months / 2nd Trimester - Week 29-delivery / 7-9 months / 3rd Trimester - Postpartum - I do not remember |

| Headache | - Yes - No |  | - Week 0-15 / 1-3 months / 1st Trimester - Week 16-28 / 4-6 months / 2nd Trimester - Week 29-delivery / 7-9 months / 3rd Trimester - Postpartum - I do not remember |
| --- | --- | --- | --- |
| Haemorrhoids and/or venous insufficiency | - Yes - No |  | - Week 0-15 / 1-3 months / 1st Trimester - Week 16-28 / 4-6 months / 2nd Trimester - Week 29-delivery / 7-9 months / 3rd Trimester - Postpartum - I do not remember |
| Sleeping problems | - Yes - No |  | - Week 0-15 / 1-3 months / 1st Trimester - Week 16-28 / 4-6 months / 2nd Trimester - Week 29-delivery / 7-9 months / 3rd Trimester - Postpartum - I do not remember |
| Gestational Diabetes | - Yes - No |  | - Week 0-15 / 1-3 months / 1st Trimester - Week 16-28 / 4-6 months / 2nd Trimester - Week 29-delivery / 7-9 months / 3rd Trimester - Postpartum - I do not remember |
| Others: | - Yes - No |  | - Week 0-15 / 1-3 months / 1st Trimester - Week 16-28 / 4-6 months / 2nd Trimester - Week 29-delivery / 7-9 months / 3rd Trimester - Postpartum - I do not remember |
| 1. **Do you suffer from any chronic pathology? □Yes □No Which one? ______________** 2. **What medication do you take for this chronic pathology? _______________________** | | | |

| 1. **During this pregnancy and/or breastfeeding, have you taken/used any herbal preparations (e.g., ginger, echinacea, valerian, cranberry, etc.)?**   **□ Yes □ No □ Cannot remember**  **If yes, use the table to answer**   - 1. **(If yes) Provide the name of all herbal preparations/products you have taken during pregnancy**   2. **(If yes) What was the reason for taking herbal preparations/products (health condition, illness)?**   3. **(If yes) In what weeks of pregnancy did you take the herbal preparations/products?** | | | |
| --- | --- | --- | --- |
| **Name of the HPs used** | **Presentation** | **Reason for use (health condition, disease)** | **Period of use (week/month/trimester of pregnancy)** |
|  | □ capsules  □ compressed   - syrup - spray - infusion - cream/pomade - others |  | - Week 0-15 / 1-3 months / 1st Trimester - Week 16-28 / 4-6 months / 2nd Trimester - Week 29-delivery / 7-9 months / 3rd Trimester - Postpartum - I do not remember |
|  | □capsules  □compressed   - syrup - spray - infusion - cream/pomade - others |  | - Week 0-15 / 1-3 months / 1st Trimester - Week 16-28 / 4-6 months / 2nd Trimester - Week 29-delivery / 7-9 months / 3rd Trimester - Postpartum - I do not remember |
|  | □capsules   - tablets - syrup - spray - infusion - cream/pomade - others |  | - Week 0-15 / 1-3 months / 1st Trimester - Week 16-28 / 4-6 months / 2nd Trimester - Week 29-delivery / 7-9 months / 3rd Trimester - Postpartum - I do not remember |
|  | □capsules   - tablets - syrup - spray - infusion - cream/pomade - others |  | - Week 0-15 / 1-3 months / 1st Trimester - Week 16-28 / 4-6 months / 2nd Trimester - Week 29-delivery / 7-9 months / 3rd Trimester - Postpartum - I do not remember |
|  | □capsules   - tablets - syrup - spray - infusion - cream/pomade - others |  | - Week 0-15 / 1-3 months / 1st Trimester - Week 16-28 / 4-6 months / 2nd Trimester - Week 29-delivery / 7-9 months / 3rd Trimester - Postpartum - I do not remember |

| **(If the answer to question 9 is yes) 10.**   1. **Who recommended you to take/use herbal preparations during pregnancy?**   (You can check more than one answer)   \| □ Self-initiative \| □ Family / friends \| \| --- \| --- \| \| □ Physician \| □ Midwife or nurse \| \| □ Pharmacist \| □ Herbalist personnel \| \| □ Nutritionist \| □ Magazines, media (TV, radio, internet) \| \| Other (specify: _________________________________________________) \| \|  1. **Have you told your physician that you are taking herbal preparations?**   □ Yes □ No □ Cannot remember | | | | | |
| --- | --- | --- | --- | --- | --- | --- | --- | --- | --- | --- | --- | --- | --- | --- | --- |
| **11. Do you follow any type of diet?**  □Yes □ No  **If yes, please indicate type of diet**   \| □ Vegetarian \| □ Flexitarian \| \| --- \| --- \| \| □ Vegan \| □ Raw \| \| □ Paleolithic \| □ Gluten free \| \| □ Hypocaloric \| □ For diabetics \| \| Others (specify: _________________________________________________) \| \| \|  \| \| | | | | | |
| **VIEWPOINT ON MEDICINES**  **We would like to ask you about your personal views on the use of**  **synthetic drugs. There are no right or wrong answers. We are interested in your opinions.** | | | | | |
| **12. These are statements that other people have made about medicines IN GENERAL. Please specify the extent to which you agree or disagree with each one by checking where appropriate (You can only check one option per line).** | | | | | |
|  | **Completely**  **disagree** | **Disagree** | **Partially**  **agree** | **Agreed** | **Completely agree** |
| Without medicines doctors would be less able to cure people | **◦** | **◦** | **◦** | **◦** | **◦** |
| Doctors recommend too many medications | **◦** | **◦** | **◦** | **◦** | **◦** |
| People who take medicines should stop their treatment for a while every now and again. | **◦** | **◦** | **◦** | **◦** | **◦** |
| Most medicines are addictive. | **◦** | **◦** | **◦** | **◦** | **◦** |
| Natural remedies are safer than medicines. | **◦** | **◦** | **◦** | **◦** | **◦** |
| Medicines do more harm than good. | **◦** | **◦** | **◦** | **◦** | **◦** |
| Medications help many people to have a better quality of life. | **◦** | **◦** | **◦** | **◦** | **◦** |
| Medications help many people to have a better quality of life. | **◦** | **◦** | **◦** | **◦** | **◦** |
| Medicines help many people to live longer. | **◦** | **◦** | **◦** | **◦** | **◦** |
| All the medicines are poisons. | **◦** | **◦** | **◦** | **◦** | **◦** |
| If physicians would spend more time with patients  would prescribe fewer drugs. | **◦** | **◦** | **◦** | **◦** | **◦** |
| In most cases, the benefits of the medications outweigh the risks. | **◦** | **◦** | **◦** | **◦** | **◦** |
| Medications improve the quality of life, but they have side effects. | **◦** | **◦** | **◦** | **◦** | **◦** |

| **13. The following are some statements on the use of medications DURING PREGNANCY. Please specify your degree of agreement or disagreement with these statements:** | | | | | |
| --- | --- | --- | --- | --- | --- |
|  | **Completely disagree** | **Disagree** | **Partially agree** | **Agreed** | **Completely agree** |
| I am more reluctant to take medication when I am pregnant than when I am not pregnant. | **◦** | **◦** | **◦** | **◦** | **◦** |
| Even if I am sick and can take medication, it is better for the fetus if I refrain from taking it. | **◦** | **◦** | **◦** | **◦** | **◦** |
| Pregnant women should preferably use herbal remedies rather than conventional drugs. | **◦** | **◦** | **◦** | **◦** | **◦** |

| **14**. **Below is a list of various medications, foods, and other substances.** Indicate how harmful you think each is to the fetus on a scale of 0 to 4, where 0 is "not harmful" and 4 is "very harmful".  If you do not know the substance/product, state "unknown substance". | | | | | | |
| --- | --- | --- | --- | --- | --- | --- |
|  | **Unknown Substance** | **0** | **1** | **2** | **3** | **4** |
| Paracetamol | **◦** | **◦** | **◦** | **◦** | **◦** | **◦** |
| Antibiotics (e.g.*Penicillin*) | **◦** | **◦** | **◦** | **◦** | **◦** | **◦** |
| Antidepressants | **◦** | **◦** | **◦** | **◦** | **◦** | **◦** |
| *Influenza* vaccine | **◦** | **◦** | **◦** | **◦** | **◦** | **◦** |
| Over-the-counter medicines for travel sickness | **◦** | **◦** | **◦** | **◦** | **◦** | **◦** |
| Ginger | **◦** | **◦** | **◦** | **◦** | **◦** | **◦** |
| Coffee | **◦** | **◦** | **◦** | **◦** | **◦** | **◦** |
| Tuna | **◦** | **◦** | **◦** | **◦** | **◦** | **◦** |
| Sausages | **◦** | **◦** | **◦** | **◦** | **◦** | **◦** |
| Cranberries | **◦** | **◦** | **◦** | **◦** | **◦** | **◦** |
| Blue Cheese (Gorgonzola) | **◦** | **◦** | **◦** | **◦** | **◦** | **◦** |
| Eggs | **◦** | **◦** | **◦** | **◦** | **◦** | **◦** |
| Alcoholic beverages | **◦** | **◦** | **◦** | **◦** | **◦** | **◦** |
| Smoking (cigarettes) | **◦** | **◦** | **◦** | **◦** | **◦** | **◦** |
| Microwave | **◦** | **◦** | **◦** | **◦** | **◦** | **◦** |
