# Supplementary material for: Herbal products use during pregnancy and postpartum: study of consumption and user profile in Catalonia
Source: BMC Complement Med Ther. 2025 Aug 8;25:301. doi: 10.1186/s12906-025-05008-4 (PMC12335089; doi:10.1186/s12906-025-05008-4)
Supplement: Supplementary file 2 — Supplementary Material 2 [file 12906_2025_5008_MOESM2_ESM.docx]

**Supplementary Material 2**

**Herbal products use during pregnancy and postpartum: Study of consumption and user profile in Catalonia.**

Noelia G. Romero ^1,2^, Elisabet Teixido ^1,2^, Laia Guardia-Escote ^1,2^, Anna Tresserra ^2,4^, Salvador Cañigueral ^3^, Marta Barenys ^1,2,5^

^1^ Unitat de Toxicologia-GRET, Departament de Farmacologia, Toxicologia i Química Terapèutica, Facultat de Farmàcia i Ciències de l’Alimentació, Universitat de Barcelona, Av. Joan XXIII 27-31, 08028 Barcelona, Spain.

^2^ Institute of Nutrition and Food Safety of the University of Barcelona (INSA-UB), Spain.

^3^ Unitat de Farmacologia, Farmacognòsia, i Terapèutica, Departament de Farmacologia, Toxicologia i Química Terapèutica, Facultat de Farmàcia i Ciències de l’Alimentació, Universitat de Barcelona, Av. Joan XXIII 27-31, 08028 Barcelona, Spain.

^4^ Polyphenol Research Group, Departament de Nutrició, Ciències de l’Alimentació i Gastronomía, Facultat de Farmàcia i Ciències de l’Alimentació, Avda. Joan XXIII, 27-31, 08028 Barcelona, Spain.

^5^ ZEBET, German Centre for the Protection of Laboratory Animals (Bf3R), German Federal Institute for Risk Assessment (BfR), Berlin, Germany.

**Questionnaire validation**


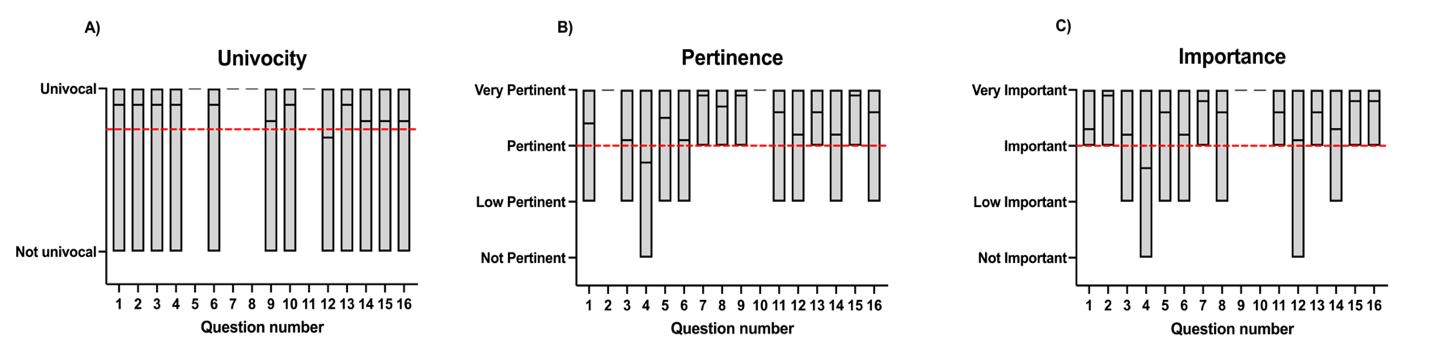
**Figure S2-1.** A box-and-whisker plot of the Exent Judgement validation of the questionnaire Kennedy et al (2013), for the parameters of univocity (A), pertinence (B) and importance (C).

According to the expert review of the (Kennedy et al., 2013) questionnaire, all questions were univocal except question 12, which obtained a mean score of 0.7 and therefore received a value below the pre-established threshold of 0.75 (on a scale of 0 to 1) for a question to be considered univocal. Following this criterion and in accordance with the experts' comments, question 12 was excluded from the questionnaire (Figure S2.1-A).

Considering that the score for pertinence and importance was between 0 and 4, questions with a median score of less than 3 on these parameters were excluded from the questionnaire, as they were considered neither pertinent nor important according to the Expert Judgement. Therefore, question 4 was excluded according to the criteria of pertinence and importance (Figure S2.1-B, C).

Following this criterion, the questions from the first questionnaire to be eliminated were questions 4 and 12, leaving the final questionnaire with 14 questions.

As a conclusion of the validation of the content of the questionnaire by Expert Judgement, the study questionnaire can be considered valid if questions 4 and 12 are removed and the comments of the experts are considered.

**Prevalence and characteristics of herbal products consumption during pregnancy and postpartum**

**
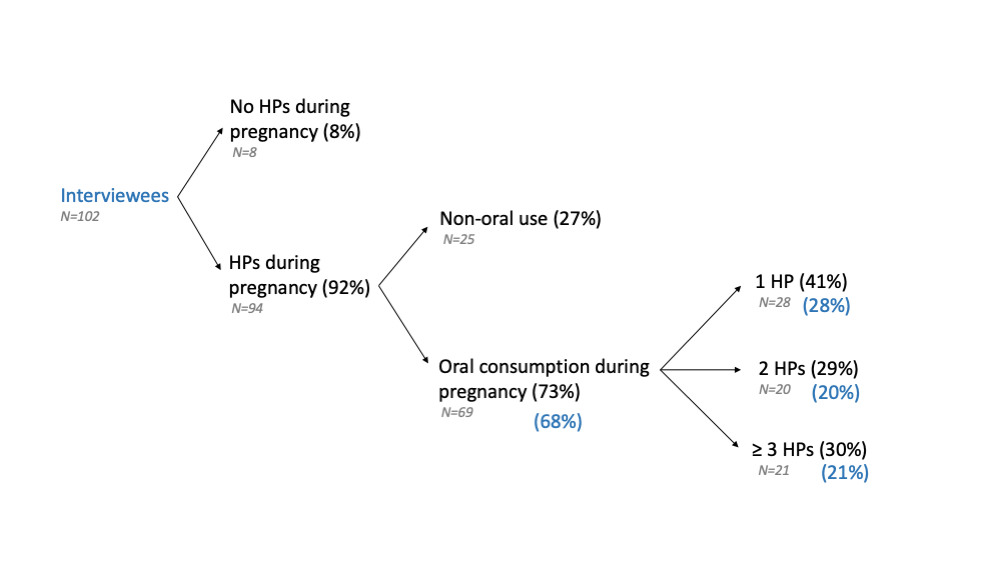
**

**Figure S2-2.** Results of consumption of HPs among the interviewees. Results consumption only during pregnancy. Black percentages are calculated based on the number of interviewees included in the group at the arrow origin (specific N indicated in gray); blue percentages are calculated based on total respondents (N=102).

**Table S2-1**. Frequency of HPs oral consumption during the 1st trimester of pregnancy.

| HPs Name | Scientific plant name^1^ | Frequency | Percentage |
| --- | --- | --- | --- |
| Ginger | *Zingiber officinale* Roscoe | 25 | 61.0 |
| Chamomile | *Matricaria chamomilla* L. | 3 | 7.3 |
| Cranberry | *Vaccinium macrocarpon* Aiton | 2 | 4.9 |
| Thyme | *Thymus vulgaris* L. / *T. zygis* L. | 2 | 4.9 |
| Rooibos | *Aspalathus linearis* (Burm. f.) R.Dahlgren. | 1 | 2.3 |
| Oregano | *Origanum vulgare* L. / *O. onites* L. | 1 | 2.3 |
| NeoBianacid* | n.a | 1 | 2.3 |
| Lemon | *Citrus limon* (L.) Osbeck | 1 | 2.3 |
| Kudzu | *Pueraria spp* | 1 | 2.3 |
| HerbalGem expectorant* | n.a | 1 | 2.3 |
| Golaplus* | n.a | 1 | 2.3 |
| Ispaghula | *Plantago ovata* Forsk. | 1 | 2.3 |
| Arkovox* | n.a | 1 | 2.3 |
| Total |  | 41 | 100 |

*Commercial herbal combinations; n.a: not applicable

**^1^**The scientific names included are those most commonly associated with the common names.

**Table S2-2.** Frequency of HPs oral consumption during the 2nd trimester of pregnancy.

| HPs Name | Scientific plant name^1^ | Frequency | Percentage |
| --- | --- | --- | --- |
| Ginger | *Zingiber officinale* Roscoe | 4 | 21.1 |
| Thyme | *Thymus vulgaris* L. / *T. zygis* L. | 4 | 21.1 |
| Echinacea | *Echinacea purpurea* (L.) Moench. / *E. angustifolia* DC. / *E. pallida* (Nutt.) Nutt. | 2 | 10.5 |
| Cranberry | *Vaccinium macrocarpon* Aiton | 1 | 5.3 |
| Floradix* | n.a | 1 | 5.3 |
| Fennel | *Foeniculum vulgare* Mill. | 1 | 5.3 |
| Cannabis leaves | *Cannabis sativa* L. | 1 | 5.3 |
| LenoDiar Aboca* | n.a | 1 | 5.3 |
| Linseed | *Linum usitatissimum* L. | 1 | 5.3 |
| NeoBianacid* | n.a | 1 | 5.3 |
| Rooibos | *Aspalathus linearis* (Burm. f.) R. Dahlgren | 1 | 5.3 |
| Lime flower | *Tilia cordata* Mill. / *T. platyphyllos* Scop., / *T.* × *europaea* L. | 1 | 5.3 |
| Total |  | 19 | 100 |

*Commercial herbal combinations; n.a: not applicable

**^1^**The scientific names included are those most commonly associated with the common names.

**Table S2-3.** Frequency of HPs oral consumption during the 3rd trimester of pregnancy.

| HPs Name | Scientific plant name^1^ | Frequency | Percentage |
| --- | --- | --- | --- |
| Raspberry leaves | *Rubus idaeus* L. | 7 | 30.4 |
| Cranberry | *Vaccinium macrocarpon* Aiton | 2 | 8.7 |
| Chamomile | *Matricaria chamomilla* L. | 2 | 8.7 |
| Ginger | *Zingiber officinale* Roscoe | 1 | 4.3 |
| Blend* |  | 1 | 4.3 |
| Floradix** | n.a | 1 | 4.3 |
| Fennel | *Foeniculum vulgare* Mill. | 1 | 4.3 |
| Yerba mate | *Ilex paraguariensis* A.St.-Hil. | 1 | 4.3 |
| NeoBianacid** | n.a | 1 | 4.3 |
| Herbal candy Ricola** | n.a | 1 | 4.3 |
| Grapefruit seed | *Citrus x paradisi* Macfad. | 1 | 4.3 |
| Som Fitotablet**  Complex** | n.a  n.a | 1 | 4.3 |
| HerbalGem Stomagen** | n.a | 1 | 4.3 |
| Lime flower | *Tilia cordata* Mill. / *T. platyphyllos* Scop., / *T.* × *europaea* L. | 1 | 4.3 |
| Thyme | *Thymus vulgaris* L. / *T. zygis* L. | 1 | 4.3 |
| Total |  | 23 | 100 |

*Lime blosoom *(Tilia cordata)*, azahar (*Citrus spp),* lemon verbena (*Aloysia citrodora* Paláu), lemon balm (*Melissa officinalis* L); ** Commercial herbal combinations; n.a: not applicable

**^1^**The scientific names included are those most commonly associated with the common names.

**Table S2-4.** Frequency of HPs oral consumption during the postpartum.

| HPs Name | Scientific plant name^1^ | Frequency | Percentage |
| --- | --- | --- | --- |
| Milk thistle | *Silybum marianum* (L.) Gaertn. | 2 | 20.0 |
| Echinacea | *Echinacea purpurea* L. Moench. / *E. angustifolia* DC. / *E. pallida* (Nutt.) Nutt. | 1 | 10.0 |
| Floradix* | n.a | 1 | 10.0 |
| Ginger | *Zingiber officinale* Roscoe. | 1 | 10.0 |
| Lemon balm | *Melissa officinalis* L. | 1 | 10.0 |
| Chamomile | *Matricaria chamomilla* L. | 1 | 10.0 |
| Hibiscus / Roselle | *Hibiscus sabdariffa*  L. | 1 | 10.0 |
| Thyme | *Thymus vulgaris* L. / *T. zygis* L. | 1 | 10.0 |
| Horsetail | *Equisetum arvense* L. | 1 | 10.0 |
| Total |  | 10 | 100 |

*Commercial herbal combinations; n.a: not applicable

**^1^**The scientific names included are those most commonly associated with the common names.

**Table S2-5.** Frequency of HPs oral consumption during the 1st and 2nd trimester of pregnancy.

| HPs Name | Scientific plant name^1^ | Frequency | Percentage |
| --- | --- | --- | --- |
| Ginger | *Zingiber officinale* Roscoe. | 2 | 18.2 |
| Cinnamon | *Cinnamomum verum* J.Presl. | 1 | 9.1 |
| Cranberry | *Vaccinium macrocarpon* Aiton | 1 | 9.1 |
| Milk thistle | *Silybum marianum* (L.) Gaertn. | 1 | 9.1 |
| Floradix* | n.a | 1 | 9.1 |
| GrinTuss* | n.a | 1 | 9.1 |
| Lemon verbena | *Aloysia citrodora* Paláu | 1 | 9.1 |
| NeoBianacid* | n.a | 1 | 9.1 |
| Rooibos | *Aspalathus linearis* (Burm. f.) R.Dahlgren | 1 | 9.1 |
| Thyme | *Thymus vulgaris* L. / *T. zygis* L. | 1 | 9.1 |
| Total |  | 11 | 100 |

*Commercial herbal combinations; n.a: not applicable

**^1^**The scientific names included are those most commonly associated with the common names.

**Table S2-6.** Frequency of HPs oral consumption during the 2nd and 3rd trimester of pregnancy.

| HPs Name | Scientific plant name^1^ | Frequency | Percentage |
| --- | --- | --- | --- |
| Chamomile | *Matricaria chamomilla* L. | 3 | 33.3 |
| Ginger | *Zingiber officinale* Roscoe. | 3 | 33.3 |
| NeoBianacid* | n.a | 2 | 22.2 |
| Fennel | *Foeniculum vulgare* Mill. | 1 | 11.1 |
| Total |  | 9 | 100 |

*Commercial herbal combinations; n.a: not applicable

**^1^**The scientific names included are those most commonly associated with the common names.

**Table S2-7.** Frequency of HPs oral consumption during the 3rd trimester of pregnancy

and postpartum.

| HPs Name | Scientific plant name^1^ | Frequency | Percentage |
| --- | --- | --- | --- |
| Red fruits* |  | 1 | 50.0 |
| Ispaghula | *Plantago ovata* Forsk. | 1 | 50.0 |
| Total |  | 2 | 100 |

*Combination of raspberry (*Rubus idaeus* L.), blueberry (*Vaccinium corymbosum* L.), strawberry *(Fragaria × ananassa)*

**^1^**The scientific names included are those most commonly associated with the common names.

**Table S2-8.** Frequency of HPs oral consumption during the 1st ,2nd and 3rd trimester

of pregnancy.

| HPs Name | Scientific plant name^1^ | Frequency | Percentage |
| --- | --- | --- | --- |
| Ginger | *Zingiber officinale* Roscoe | 7 | 22.6 |
| Chamomile | *Matricaria chamomilla* L | 5 | 16.1 |
| Rooibos | *Aspalathus linearis* (Burm. f.) R.Dahlgren | 5 | 12.9 |
| Anisee  Tea* | *Pimpinella anisum* L.  *Camellia sinensis* (L.) Kuntze | 2  2 | 6.5  6.5 |
| Blend** |  | 1 | 3.2 |
| Cranberry | *Vaccinium macrocarpon* Aiton | 1 | 3.2 |
| Floradix*** | n.a | 1 | 3.2 |
| GaviNatura*** | n.a | 1 | 3.2 |
| Yerba mate | *Ilex paraguariensis* A. St.-Hil. | 1 | 3.2 |
| Lemon | *Citrus limon* (L.) Osbeck | 1 | 3.2 |
| NeoBianacid*** | n.a | 1 | 3.2 |
| Pennyroyal | *Mentha pulegium* L. | 1 | 3.2 |
| Thyme | *Thymus vulgaris* L. / *T. zygis* L. | 1 | 3.2 |
| Valerian | *Valeriana officinalis* L. | 1 | 3.2 |
| Total |  | 31 | 100 |

*black, red or green, **ginger (*Zingiber officinale* Roscoe), licorice (*Glycyrrhiza glabra* L*.* / *G. inflata* Batalin / *G. uralensis* Fisch.*),* peppermint (*Mentha piperita* L); *** Commercial herbal combinations; n.a: not applicable

**^1^**The scientific names included are those most commonly associated with the common names.

**Table S2-9.** Frequency of HPs oral consumption during the 1st ,2nd trimester of pregnancy

and postpartum.

| HPs Name | Scientific plant name^1^ | Frequency | Percentage |
| --- | --- | --- | --- |
| Ginger | *Zingiber officinale* Roscoe | 1 | 100.0 |

**^1^**The scientific names included are those most commonly associated with the common names.

**Table S2-10.** Frequency of HPs oral consumption during 1st ,2nd ,3rd trimester of pregnancy

and postpartum.

| HPs Name | Scientific name^1^ | Frequency | Percentage |
| --- | --- | --- | --- |
| Rooibos | *Aspalathus linearis* (Burm. f.) R.Dahlgren. | 3 | 21.4 |
| Chia | *Salvia hispanica* L. | 2 | 14.3 |
| Curcumin | Curcumin is a constituent of *Curcuma longa* L. / *C. zanthorrhiza* Roxb. | 2 | 14.3 |
| Ginger  Boldo | *Zingiber officinale* Roscoe.  *Peumus boldus* Molina. | 1  1 | 7.1  7.1 |
| Chamomile | *Matricaria chamomilla* L. | 1 | 7.1 |
| Horsetail | *Equisetum arvense* L. | 1 | 7.1 |
| Licorice | *Glycyrrhiza glabra* L. / *G. inflata* Batalin / *G. uralensis* Fisch. | 1 | 7.1 |
| Linseed | *Linum usitatissimum* L. | 1 | 7.1 |
| Thyme | *Thymus vulgaris* L. / *T. zygis* L. | 1 | 7.1 |
| Total |  | 14 | 100 |

**^1^**The scientific names included are those most commonly associated with the common names.

**Table S2-11.** Frequency of HPs oral consumption, considering that participants

could not remember the period during which they consumed them.

| HPs Name | Scientific plant name^1^ | Frequency | Percentage |
| --- | --- | --- | --- |
| Ginger | *Zingiber officinale* Roscoe | 1 | 25.0 |
| Horsetail | *Equisetum arvense* L | 1 | 25.0 |
| Pranarom calming* | n.a | 1 | 25.0 |
| Tea** | *Camellia sinensis* (L.) Kuntze. | 1 | 25.0 |
| Total |  | 4 | 100 |

*Commercial herbal combinations; ** black, red or green, n.a: not applicable

**^1^**The scientific names included are those most commonly associated with the common names.

**Table S2-12.** Main herbal drugs present in commercial herbal combinations consumed orally.

| Commercial herbal combinations | Producer | Main plant drugs |
| --- | --- | --- |
| Arkovox | Arkopharma | marshmallow, papain from papaya and lemon. |
| Floradix | Salus | spinach leaves, nettle leaves, fennel fruits, carrot, centaury herb and dry extract of rose hips. |
| GaviNatura | Reckitt Benckiser | aloe vera, mallow and chamomile. |
| Grintuss | Aboca | gumweed herb, ribwort plantain, sandy everlasting, eucalyptus, star anise and lemon. |
| Herbal candy Ricola | Ricola | melissa, white horehound, pimpernel, veronica, marshmallow, dandelion, elder, mallow, mint, sage, yarrow, primrose, and ribwort plantain. |
| HerbalGem Expectorante | Pranarom | elder, purple conflower, rosemary, elecampane, white horehound, hyssop, alder, pino and myrtle QT cineol. |
| LenoDiar Aboca | Aboca | agrimony, tormentil, curcumin and olive. |
| NeoBianacid | Aboca | aloe vera, marshmallow, mallow, chamomile and licorice |
| Pranarom Calming | Pranarom | lemon, peppermint, camphor, thyme, oregano, cinnamom, laurel and clove. |
| Som Fitotablet Complex | Eladiet | valerian, passionflower, hawthorn and hop. |
| Stomagem | Pranarom | lemon, elder, fig treea and rosemary. |

**Table S2-13.** HPs orally most consumed during pregnancy and postpartum.

| Herbal Products | Producer | Frequency ^a^ | Percentage |
| --- | --- | --- | --- |
| Ginger | Arkopharma, Natura Essenziale, unknown | 46 | 28.0 |
| Chamomile | unknown | 15 | 9.1 |
| Thyme | unknown | 11 | 6.7 |
| Rooibos | unknown | 10 | 6.1 |
| Cranberry | Arkopharma, Solaray, Uriach, Salvat, Pierre Fabre, unknown | 7 | 4.3 |
| Raspberry leaf | unknown | 7 | 4.3 |
| NeoBianacid* | Aboca | 7 | 4.3 |
| Floradix* | Salus | 5 | 3.0 |
| Milk thistle | DietiNatura, Humana | 3 | 1.8 |
| Fennel | unknown | 3 | 1.8 |
| Tea (black, red, green)  Horsetail | unknown  unknown | 3  3 | 1.8  1.8 |
| Blend** | unknown | 2 | 1.2 |
| Aniseed  Boldo  Chia | unknown  unknown  unknown | 2  2  2 | 1.2  1.2  1.2 |
| Curcumin | unknown | 2 | 1.2 |
| Yerba mate | unknown | 2 | 1.2 |
| Lemon | unknown | 2 | 1.2 |
| Ispaghula  Linseed  Echinacea  Lime blosoom | Rottapharm Madaus  unknown  Rottapharm Madaus, unknown  unknown | 2  2  2  2 | 1.2  1.2  1.2  1.2 |
| Arkovox* | Arkopharma | 1 | 0.6 |
| Cinnamon | unknown | 1 | 0.6 |
| Red fruits | unknown | 1 | 0.6 |
| GaviNatura* | Reckitt Benckiser | 1 | 0.6 |
| GolaFred* | Santiveri | 1 | 0.6 |
| Grintuss* | Aboca | 1 | 0.6 |
| HerbalGem Expectorante* | Pranarom | 1 | 0.6 |
| Hibiscus | unknown | 1 | 0.6 |
| Cannabis leaves | unknown | 1 | 0.6 |
| Kudsú | unknown | 1 | 0.6 |
| LenoDiar Aboca* | Aboca | 1 | 0.6 |
| Lemon verbena | unknown | 1 | 0.6 |
| Melissa | unknown | 1 | 0.6 |
| Oregano | unknown | 1 | 0.6 |
| Herbal candy Ricola* | Ricola | 1 | 0.6 |
| Pennyroyal mint | unknown | 1 | 0.6 |
| Pranarom Calming* | Pranarom | 1 | 0.6 |
| Licorice | unknown | 1 | 0.6 |
| Grapefruit seed | unknown | 1 | 0.6 |
| Som Fitotablet Complex* | Eladiet | 1 | 0.6 |
| Stomagem* | Pranarom | 1 | 0.6 |
| Valerian  Galega | unknown  Humana | 1  1 | 0.6  0.6 |
| Total |  | **165** | **100** |
| ^a^ Number of HPs consumed orally |  |  |  |
|  | | | |

**
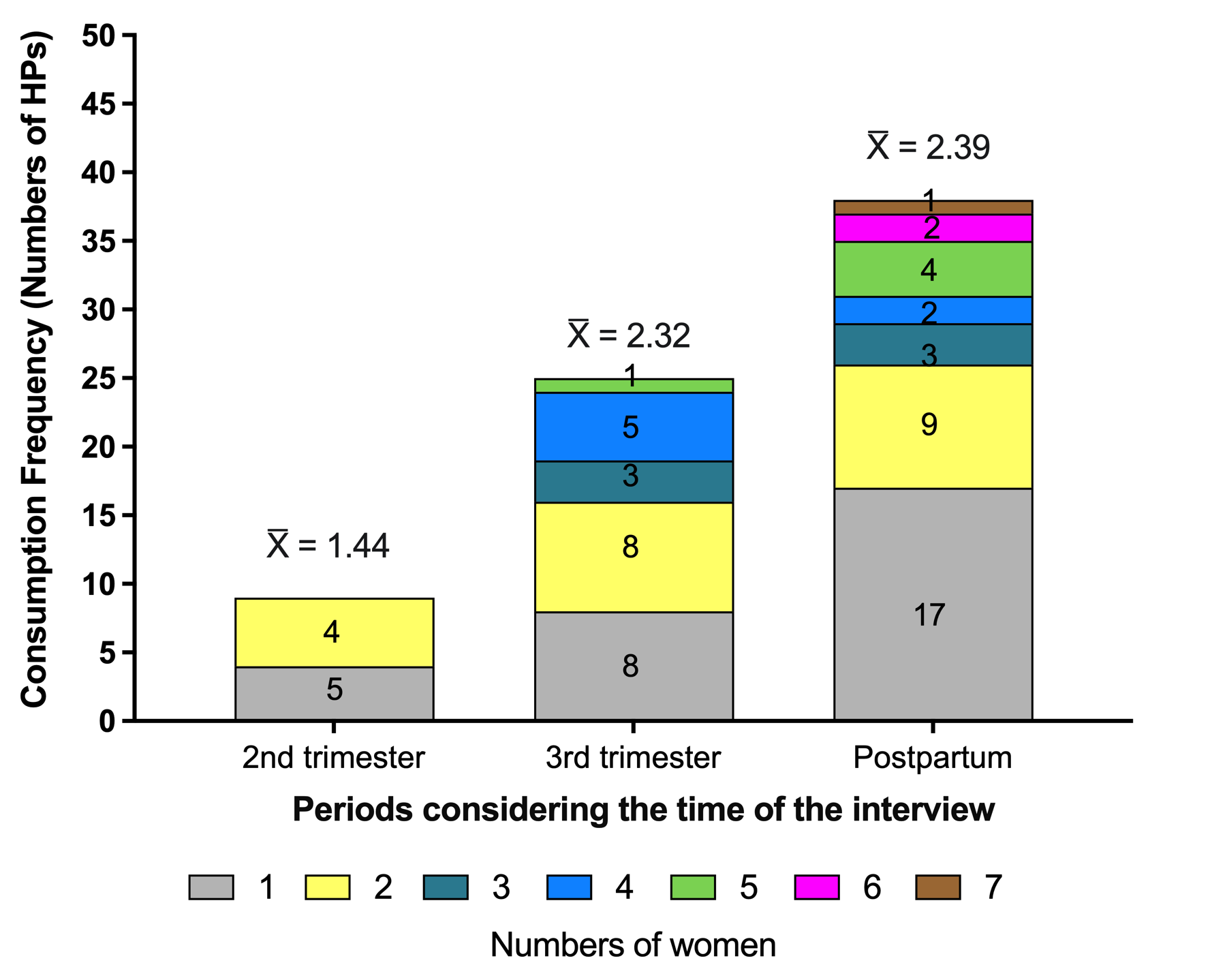
**

**Figure S2-3.** Relationship between the number of women and the number of HPs consumed

orally at the time of the interview. The different colours indicate the number of HPs consumed (1-7) and the number in each colour indicates the number of women who consumed.The mean (x̅) of HPs consumed according to the time of interview is presented: 2nd trimester (x̅ = 1.44), 3rd trimester (x̅ = 2.32) and postpartum period (x̅ = 2.39). Considering the time of the interview (1st, 2nd trimester and postpartum), the Anova of the mean (x̅) of orally consumed HPs was non-significant (p=0.229).

**Table S2-14.** Motives or reasons for use of orally consumed HPs.

| Motives or reasons for oral use  of HPs | Frequency ^a^ | Percentage |
| --- | --- | --- |
| Nausea / Upset stomach / Gastric distress | 34 | 20.6 |
| Part of the diet | 21 | 12.7 |
| Cold (prevention and treatment) | 18 | 10.9 |
| Stomach acidity | 11 | 6.7 |
| Pain (various) | 7 | 4.2 |
| Relaxing | 7 | 4.2 |
| Preparing for childbirth | 6 | 3.6 |
| Stomach reflux | 6 | 3.6 |
| Coffee substitute | 6 | 3.6 |
| Constipation | 5 | 3.0 |
| Urinary tract infection (prevention / treatment) | 5 | 3.0 |
| Insomnia + sleeping problems | 5 | 3.0 |
| Vitamin supplementation | 4 | 2.4 |
| Heartburn and stomach reflux | 3 | 1.8 |
| Breast milk production | 3 | 1.8 |
| Detoxification | 2 | 1.2 |
| Digestive | 2 | 1.2 |
| Nausea + Part of the diet | 2 | 1.2 |
| Consumed for its anti-inflammatory and diuretic properties | 2 | 1.2 |
| Postpartum Disinfection | 2 | 1.2 |
| Aphonia | 1 | 0.6 |
| Diuretic | 1 | 0.6 |
| Hormone balance and fertility | 1 | 0.6 |
| Intestinal gas | 1 | 0.6 |
| Dizziness | 1 | 0.6 |
| Menopause | 1 | 0.6 |
| Nausea + Insomnia | 1 | 0.6 |
| Part of the diet + Muscle pain | 1 | 0.6 |
| Hepatic protector | 1 | 0.6 |
| Increased defences | 1 | 0.6 |
| Candidiasis | 1 | 0.6 |
| Ferric supplementation | 1 | 0.6 |
| Coffee substitute + relaxant | 1 | 0.6 |
| Toning of the uterus | 1 | 0.6 |
| Total | 165 | 100.0 |
| ^a^ Number of HPs consumed orally. |  |  |
