# Supplementary material for: Herbal products use during pregnancy and postpartum: study of consumption and user profile in Catalonia
Source: BMC Complement Med Ther. 2025 Aug 8;25:301. doi: 10.1186/s12906-025-05008-4 (PMC12335089; doi:10.1186/s12906-025-05008-4)
Supplement: Supplementary file 3 — Supplementary Material 3 [file 12906_2025_5008_MOESM3_ESM.docx]

**Supplementary Material 3**

**Herbal products use in Catalonia during pregnancy and postpartum: Study of consumption and user profile.**

Noelia G. Romero ^1,2^, Elisabet Teixido ^1,2^, Laia Guardia-Escote ^1,2^, Anna Tresserra ^2,4^, Salvador Cañigueral ^3^, Marta Barenys ^1,2,5^

^1^ Unitat de Toxicologia-GRET, Departament de Farmacologia, Toxicologia i Química Terapèutica, Facultat de Farmàcia i Ciències de l’Alimentació, Universitat de Barcelona, Av. Joan XXIII 27-31, 08028 Barcelona, Spain.

^2^ Institute of Nutrition and Food Safety of the University of Barcelona (INSA-UB), Spain.

^3^ Unitat de Farmacologia, Farmacognòsia, i Terapèutica, Departament de Farmacologia, Toxicologia i Química Terapèutica, Facultat de Farmàcia i Ciències de l’Alimentació, Universitat de Barcelona, Av. Joan XXIII 27-31, 08028 Barcelona, Spain.

^4^ Polyphenol Research Group, Departament de Nutrició, Ciències de l’Alimentació i Gastronomía, Facultat de Farmàcia i Ciències de l’Alimentació, Avda. Joan XXIII, 27-31, 08028 Barcelona, Spain.

^5^ ZEBET, German Centre for the Protection of Laboratory Animals (Bf3R), German Federal Institute for Risk Assessment (BfR), Berlin, Germany.

**Profile of HP oral consumers**

| **Variables in the Equation** | **B estimated coefficient** | **Standard Error** | **Wald statistic** | **Degrees of freedom** | **p value** | **Exp (B) Odds ratio** | **Confidence interval (95%)** |
| --- | --- | --- | --- | --- | --- | --- | --- |
| Recruitment centres: public or private | 0.311 | 0.473 | 0.433 | 1 | 0.511 | 0.733 | 0.290 – 1.851 |
| Health Region | 0.650 | 0.230 | 8.018 | 1 | **0.005** | 1.915 | 1.221 – 3.003 |
| Country of birth | 0.099 | 0.244 | 0.163 | 1 | 0.686 | 1.104 | 0.684 – 1.781 |
| Pregnant or in the postpartum period | 0.738 | 0.542 | 1.855 | 1 | 0.173 | 0.478 | 0.165 – 1.383 |
| Children before this pregnancy | 0.590 | 0.568 | 1.078 | 1 | 0.299 | 0.554 | 0.182 – 1.689 |
| Employment status when she became pregnant | 0.456 | 0.261 | 3.040 | 1 | 0.081 | 0.634 | 0.380 – 1.058 |
| Age | 0.038 | 0.070 | 0.297 | 1 | 0.586 | 0.962 | 0.839 – 1.104 |
| Highest level of education | 0.725 | 0.491 | 2.183 | 1 | 0.140 | 0.484 | 0.185 – 1.267 |
| Suffering from chronic pathology | 0.102 | 0.611 | 3.258 | 1 | 0.071 | 3.010 | 0.910 – 9.961 |
| Consumption of medicines | 1.611 | 0.677 | 5.657 | 1 | **0.017** | 5.009 | 1.328 – 18.894 |

**Table S3-1.** Personal and socio-demographic variables related to oral HP use.

Significance threshold established at p ≤ 0.05

**Consumers opinions on medicines in general and during pregnancy**

For the analysis of perceptions of medicine use in general, the questions (based on the questions included in the questionnaire of Kennedy et al., 2013) have been classified into the following categories: Extreme and Not extreme; and Positive and Negative towards medical drugs:

**Table S3-2.** Questions related to the use of conventional medicines in general have been classified as Extreme and Not extreme.

| Extreme | Not Extreme |
| --- | --- |
| Without medicines, doctors would be less able to cure people. | Natural remedies are safer than drugs. |
| Doctors recommend too many medicines. | Medicines help many people to have a better quality of life. |
| People who take medicines should stop their treatment for a while from time to time. | Medicines help many people to live longer. |
| Most medicines are addictive. | If doctors had more time with patients they would prescribe fewer medicines. |
| Medicines cause more harm than good. | The benefits of medicines outweigh the risks in most cases. |
| All medicines are poisons. | Medicines improve quality of life, but have side effects. |
| Doctors rely too much on medicines. |  |

**Table S3-3.** Questions related to the use of conventional medicines in general have been classified as Positive and Negative towards medical drugs.

| Positive | Negative |
| --- | --- |
| Without medicines, doctors would be less able to cure people. | Doctors recommend too much medication. |
| Medicines help many people to live a better quality of life. | People taking medicines should stop their treatment for a while from time to time. |
| Medicines help many people to live longer. | Most medicines are addictive. |
| Medicines improve quality of life but have side-effects. | Natural remedies are safer than drugs. |
|  | Medicines cause more harm than good. |
|  | All medicines are poisons. |
|  | Doctors rely too much on medicines. |
|  | If doctors had more time with patients they would prescribe less drugs. |
|  | In most cases, the benefits of drugs outweigh the risks. |

**Table S3-4.** Opinions of the women interviewees on the safety of ginger for the foetus.

Answer options: Not harmful, Not very harmful, Partially harmful, Harmful, Very harmful,

Unknown substance.

| Perception of safety for the foetus* | Frequency | Percent |
| --- | --- | --- |
| Not harmful | 58 | 57 |
| Not very harmful | 31 | 30 |
| Partially harmful | 7 | 7 |
| Harmful  Very harmful  Unknown substance | 4  1  1 | 4  1  1 |
| Total | **102*** | **100** |

** Based on total number of respondents (N=102).*

**Table S3-5.** Opinions of the women interviewees on the safety of cranberry for the foetus.

Answer options: Not harmful, Not very harmful, Partially harmful, Harmful, Very harmful,

Unknown substance.

| Perception of safety for the foetus | Frequency | Percent |
| --- | --- | --- |
| Not harmful | 65 | 64 |
| Not very harmful | 19 | 18 |
| Partially harmful | 3 | 3 |
| Harmful  Unknown substance | 3  12 | 3  12 |
| Total | **102*** | **100** |

** Based on total number of respondents (N=102).*


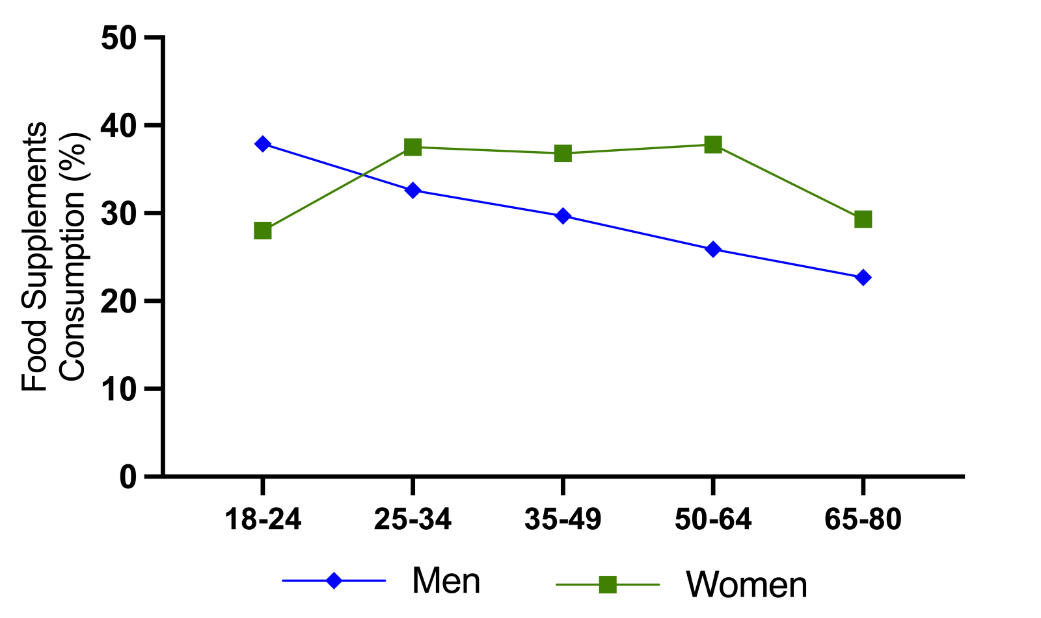


**Figure S3-1.** Frequency of consumption (%) of food supplements in the

Catalan population by age range and sex. The data correspond to the technical

sheet called Food Safety Barometer 2022 of the Catalan Food Safety Agency

of the Government of Catalonia (Agència Catalana de Seguretat Alimentària, 2022).
